# Aspherical atom refinements on X-ray data of diverse structures including disordered and covalent organic framework systems: a time–accuracy trade-off

**DOI:** 10.1107/S1600576722010883

**Published:** 2023-02-01

**Authors:** Kunal Kumar Jha, Florian Kleemiss, Michał Leszek Chodkiewicz, Paulina Maria Dominiak

**Affiliations:** a Biological and Chemical Research Centre, Department of Chemistry, University of Warsaw, ul. Żwirki i Wigury 101, Warsaw, 02-089, Poland; b Fakultät für Chemie und Pharmazie, Universität Regensburg, Universitätstrasse 31, Regensburg, Bayern 93053, Germany; SLAC National Accelerator Laboratory, Menlo Park, USA

**Keywords:** quantum crystallography, aspherical atom refinement, transferable aspherical atom model, TAAM, MATTS, Hirshfeld atom refinement, HAR, *NoSpherA2*, disorder, structure refinement

## Abstract

Aspherical atom refinement is the key to achieving accurate structure models, displacement parameters, hydrogen-bond lengths and analysis of weak interactions, amongst other examples. There are various quantum crystallographic methods to perform aspherical atom refinement, including Hirshfeld atom refinement (HAR) and transferable aspherical atom model (TAAM) refinement. Both HAR and TAAM have their limitations and advantages, the former being more accurate and the latter being faster. With the advent of non-spherical atoms in *Olex2* (*NoSpherA2*), it is now possible to overcome some limitations, like treating disorder, twinning and network structures, in aspherical refinements using HAR, TAAM or both together. TAAM refinement in *NoSpherA2* showed significant improvement in refinement statistics compared with independent atom model (IAM) refinements on a diverse set of X-ray diffraction data. The sensitivity of TAAM towards poor data quality and disorder was observed in terms of higher refinement statistics for such structures. A comparison of IAM with TAAM and HAR in *NoSpherA2* indicated that the time taken by TAAM refinements was of the same order of magnitude as that taken by IAM, while in HAR the time taken using a minimal basis set was 50 times higher than for IAM and rapidly increased with increasing size of the basis sets used. The displacement parameters for hydrogen and non-hydrogen atoms were very similar in both HAR and TAAM refinements. The hydrogen-bond lengths were slightly closer to neutron reference values in the case of HAR with higher basis sets than in TAAM. To benefit from the advantages of each method, a new hybrid refinement approach has been introduced, allowing a combination of IAM, HAR and TAAM in one structure refinement. Refinement of coordination complexes involving metal–organic compounds and network structures such as covalent organic frameworks and metal–organic frameworks is now possible in a hybrid mode such as IAM–TAAM or HAR–TAAM, where the metal atoms are treated via either the IAM or HAR method and the organic part via TAAM, thus reducing the computational costs without compromising the accuracy. Formal charges on the metal and ligand can also be introduced in hybrid-mode refinement.

## Abstract 2

It is now possible to integrate the transferable aspherical atom model (TAAM) with *NoSpherA2* and refine X-ray diffraction data of disordered, twinned, co-crystal, covalent organic framework and metal–salt structures in a short period of time. A new hybrid approach, allowing a combination of the independent atom model, Hirshfeld atom refinement and TAAM in one structure refinement, is introduced which benefits from the advantages of each method.

## 1. Introduction

The recent advances in quantum crystallography have facilitated a shift from the common but approximate independent atom model (IAM) used in structure determination to a more appropriate aspherical atom model (Jelsch *et al.*, 2005 ▸; Dittrich *et al.*, 2006 ▸, 2008 ▸; Wandtke *et al.*, 2017 ▸; Woińska *et al.*, 2016 ▸; Jayatilaka & Dittrich, 2008 ▸; Malaspina *et al.*, 2019 ▸; Volkov *et al.*, 2007 ▸; Hoser *et al.*, 2009 ▸; Malinska & Dauter, 2016 ▸; Kumar *et al.*, 2014 ▸; Jha *et al.*, 2020 ▸; Lübben *et al.*, 2019 ▸; Bergmann *et al.*, 2020 ▸; Sanjuan-Szklarz *et al.*, 2020 ▸). With IAM, which is based on scattering factors precomputed for isolated, spherically averaged, non-interacting atoms or ions, the structural information is limited to nucleus position only (Coppens, 1997 ▸). This model has been adopted for years due to its simplicity and the resulting convenience of use and quick output. However, there are many shortfalls associated with IAM, such as a loss of information about bonding between the atoms or lone pairs, incorrect positioning of light atoms like hydrogen *etc*., leading to physically meaningless properties (Stalke, 2012 ▸).

Asphericity was experimentally observed by X-ray diffraction from the very beginning (Bragg, 1920 ▸; Franklin, 1950 ▸). Asphericity in atoms arises from the fact that atoms are bonded covalently or non-covalently and the electrons are shared, pulled/pushed towards each other, depending on the electronegativity of the interacting atoms. The impact of such events can only be revealed when the atoms are refined aspherically (Stewart *et al.*, 1965 ▸; Coppens, 1968 ▸, 1997 ▸; Hansen & Coppens, 1978 ▸; Ewald & Hönl, 1936 ▸; McWeeny, 1951 ▸; Dawson, 1967 ▸; Stewart, 1969 ▸, 1976 ▸; Hirshfeld, 1971 ▸). Aspherical refinements lead to more accurate and precise information on chemical bonding, non-covalent interactions, lone pairs, partial charges, hydrogen-atom positions *etc*. (Munshi & Guru Row, 2005 ▸; Dittrich *et al.*, 2017 ▸; Hoser *et al.*, 2009 ▸; Stalke, 2012 ▸). However, a lot of experimental or computational effort is required to get such information with high accuracy. The asphericity can be modelled using experimental high-resolution data (with the minumum interplanar spacing *d* < 0.5 Å) by refining core and valence electron populations and expansion–contraction parameters via the Hansen and Coppens multipole model of electron charge density (Hansen & Coppens, 1978 ▸). However, such aspherical models require many additional parameters to be refined and hence highly accurate, redundant and high-resolution data sets are necessary. As well as the long data collection time, experimental data sets are often still of insufficient quality for full charge-density refinements. Parametrization of aspherical models from experimental data is a very time-consuming and tedious process. Moreover, there are cases of disorder that are almost impossible to model on the basis of charge-density refinements using experimental data.

The computational efforts have been focused on achieving highly accurate aspherical models and at the same time on reducing the computational cost. Hirshfeld atom refinement (HAR) (Hirshfeld, 1977 ▸; Jayatilaka & Dittrich, 2008 ▸; Capelli *et al.*, 2014 ▸) achieves high accuracy and precision but requires the calculation of wavefunctions, which takes significantly longer than IAM. HAR combined with extremely localized molecular orbitals (ELMOs) (Meyer *et al.*, 2016 ▸), together called HAR–ELMO (Malaspina *et al.*, 2019 ▸), reduces the computational time. HAR–ELMO is based on the transferability of ELMOs; however, its use is restricted by the limited availability of precomputed molecular orbitals.

A convenient and computationally cheaper approach was developed in which a transferable aspherical atom model (TAAM) was used for the refinement (Pichon-Pesme *et al.*, 1995 ▸; Volkov *et al.*, 2004 ▸; Dominiak *et al.*, 2007 ▸; Zarychta *et al.*, 2007 ▸; Dittrich *et al.*, 2013 ▸). TAAM uses sets of multipole parameters taken from a databank. Atoms with similar chemical environments and hybridization have similar multipole parameters, and this information can be stored in a databank for parametrization of similar atoms present in a new structure. The three most popular databanks available are ELMAM2, Invariom and UBDB, the later superseded by MATTS (Domagała *et al.*, 2012 ▸; Dittrich *et al.*, 2006 ▸, 2013 ▸; Dominiak *et al.*, 2007 ▸; Jarzembska *et al.*, 2012 ▸; Kumar *et al.*, 2019 ▸; Jha *et al.*, 2022 ▸). The advantage of using such a databank transfer approach is that the time taken in the refinement is comparable to that of using IAM. TAAM refinements have been applied to both X-ray and electron diffraction (ED) data, largely improving the physical representation and refinement statistics of the crystal structures (Bąk *et al.*, 2011 ▸; Jha *et al.*, 2020 ▸; Gruza *et al.*, 2020 ▸). With non-spherical atoms in *Olex2* (*NoSpherA2*) (Kleemiss *et al.*, 2021 ▸), it is now possible to fully integrate TAAM with *NoSpherA2* and perform the refinement on X-ray and ED data of even disordered structures in a short period of time, which was not otherwise possible in an automatic way with any databank transfer approach. Within *NoSpherA2*, TAAM can also be used for hybrid refinement together with IAM and HAR. Hybrid refinement of a protein structure bound with inhibitor was shown earlier by Guillot *et al.* (2008 ▸), where the inhibitor part was refined using IAM and the protein part was refined aspherically using TAAM. The idea of a hybrid refinement using IAM for the metal and an aspherical model for the organic ligand in a coordination compound was presented by Dittrich *et al.* (2015 ▸) and Wandtke *et al.* (2017 ▸) to resolve the ambiguity of the type of the central metal atom in deposited structures. This can further be utilized for more diverse structures such as metal–organic frameworks (MOFs) or multi-component structures using an IAM/HAR/TAAM hybrid approach.

In this paper we highlight the possibility of TAAM refinement for organic molecules, co-crystals, and disordered, twinned, network and polymeric structures such as covalent organic frameworks (COFs) *etc*. using *NoSpherA2*. TAAM refinement was performed using the MATTS2021 databank (Jha *et al.*, 2022 ▸), which was built upon the restructuring and extension of UBDB2018 (Kumar *et al.*, 2019 ▸). TAAM refinement is comparatively quicker; however, HAR gives slightly better refinement statistics and hydrogen-bond distances, as discussed in an earlier report (Jha *et al.*, 2020 ▸). Here we have used the density functional theory approach with a series of basis sets and the same functional to compute the scattering factors for HAR and compared the refinement statistics as well as the time taken in each calculation with the TAAM refinement in *NoSpherA2* on selected model mol­ecules. This comparison aims to highlight the use of an aspherical model even on structures with poor data quality and moderate resolution for achieving a better structural model. Finally, we introduce a hybrid IAM/TAAM/HAR refinement, where various chemical moieties including MOFs can be parametrized using different scattering models to speed up the procedure or to overcome the lack of proper parametrization in TAAM or HAR.

## 2. Methodology

### 2.1. Implementation

The *DiSCaMB* library, capable of calculating scattering factors for the Hansen–Coppens multipole model (Chodkiewicz *et al.*, 2018 ▸), was extended to support TAAM by adding the capability to recognize atom types and handle TAAM parameters from the MATTS2021 pseudoatom databank. The details of the atom-typing algorithm implementation and new databank format MATTS2021 have been published elsewhere (Jha *et al.*, 2022 ▸). The *DiSCaMB* library was further integrated into *NoSpherA2*.

For this study, we have used *NoSpherA2* in *Olex2-1.5* (Dolomanov *et al.*, 2009 ▸) for the refinements. It incorporates the TAAM scattering factors available in the *DiSCaMB* library into the *olex2.refine* module and permits the use of these aspherical atomic form factors via a text file in the .tsc file format (Kleemiss *et al.*, 2021 ▸). The aspherical atomic form factors in the .tsc file are used during the least-squares refinement against experimental intensities in an iterative cycle until convergence is achieved. The *DiSCaMB* component necessary to run TAAM refinements under *NoSpherA2* can be downloaded from http://4xeden.uw.edu.pl/.

### 2.2. Crystal structures used for the TAAM refinement

**Figure 1:**
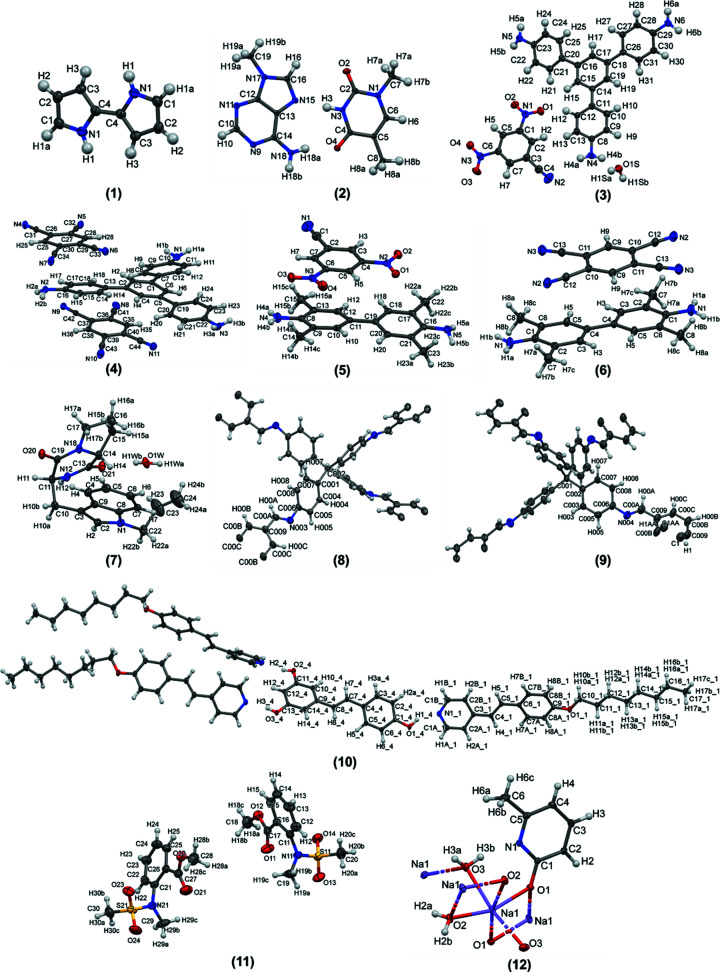
Visualization of structures used for spherical and aspherical refinement in this study. Atom labels are shown on the structures; the same atom labels indicate the symmetry-generated atoms. In COF structures **8** and **9**, only the atoms in the asymmetric unit are labelled for clarity. In structure **10**, out of the three identical molecules in the asymmetric unit, atoms of only one molecule are shown labelled.

A diverse set of already published crystal structures was chosen for this study, which included small organic molecules, co-crystals, multi-component systems, disordered structures, COFs and twinned structures (Fig. 1 ▸). Structure **1** [molecular formula (MF) = C_8_H_8_N_2_] is a simple organic system and contains half a molecule in the asymmetric unit (Michaels *et al.*, 2017 ▸). Structure **2** (MF = C_12_H_15_N_7_O_2_) is a co-crystal (Jarzembska *et al.*, 2013 ▸). In cases **1** and **2** the reported data set contained only merged reflections. Structures **3**–**6** are two-component systems (Bhowal *et al.*, 2021 ▸) having MFs C_31_H_26_N_6_O_5_ (**3**), C_44_H_25_N_11_ (**4**), C_23_H_23_N_5_O_4_ (**5**) and C_26_H_22_N_6_ (**6**). In these cases, data collected at two different temperatures (100 and 300 K) were also considered to highlight the effect of temperature on the refinement statistics. Structure **6** has half a molecule of both components in the asymmetric unit. Structure **7** (MF = C_19_H_23_N_3_O_3_) shows disorder in a terminal alkyl chain; the disorder was not modelled in the reported structure (Yi-Hua *et al.*, 2019 ▸). Structures **8** and **9** are COF systems with masked solvent disorder (Ma *et al.*, 2018 ▸); in the case of **9** there is also disorder in the ring. Structure **10** (MF = C_77_H_93_N_3_O_6_) is a hydrogen-bonded liquid crystal based on resveratrol having two different components in a 3:1 ratio in the asymmetric unit (Blanke *et al.*, 2020 ▸). Structure **11** (MF = C_20_H_26_N_2_O_8_S_2_) is twinned and contains two molecules in the asymmetric unit (Shafiq *et al.*, 2009 ▸). Structure **12** (MF = C_6_H_10_NNaO_3_) is a network alkali metal coordination complex with organic molecules (Clegg & Tooke, 2013 ▸). The coordinated metal–organic complex structure **12** represents an ideal case for hybrid IAM/HAR/TAAM refinement.

### 2.3. Refinement process using IAM

The initial geometries taken from the published structures were re-refined using the default IAM framework (Wilson & Geist, 1993 ▸) with *olex2.refine* in *Olex2-1.5* (Dolomanov *et al.*, 2009 ▸) without making any changes in resolution limit or diffraction data. Hydrogen atoms were refined with isotropic displacement parameters freely without any restraints or constraints. The most commonly used spherical density model for bonded hydrogen in the IAM approach recommended by Stewart *et al.* (1965 ▸) was used for hydrogen-atom refinement. The published results from *SHELXL* (Sheldrick, 2015 ▸) IAM refinement were used for comparison.

### 2.4. Refinement process using TAAM in *NoSpherA2*

The structures obtained from IAM were further refined with *olex2.refine* using the TAAM approach and MATTS databank (Jha *et al.*, 2022 ▸). The *DiSCaMB* library (Chodkiewicz *et al.*, 2018 ▸) integrated into *NoSpherA2* (Kleemiss *et al.*, 2021 ▸) was used for the transfer of aspherical atomic form factors into the .tsc file format (Kleemiss *et al.*, 2021 ▸). Hydrogen atoms were refined with isotropic displacement parameters freely without any restraints or constraints. The maximum number of iterative cycles was set to ten in *NoSpherA2* for the TAAM refinement; however, convergence was usually achieved after four to five cycles.

### 2.5. Refinement process using HAR in *NoSpherA2*

The structures obtained from IAM were also refined with *olex2.refine* using HAR in *NoSpherA2*. *ORCA* version 4.2.1 was used for wavefunction calculation (Neese, 2012 ▸). HAR in *NoSpherA2* was performed using the B3LYP functional (Lee *et al.*, 1988 ▸) and various basis sets. Hydrogen atoms were refined with isotropic displacement parameters freely without any restraints or constraints. The integration accuracy, self-consistent field (SCF) threshold and SCF strategy (convergence) were set to normal. Ten iterative cycles were set in the *NoSpherA2* setting. The calculations were performed on a laptop computer with a single-node 64-bit Intel i7 processor with four cores running at 2.60 GHz and 16 GB RAM.

### 2.6. Refinement process using hybrid-mode IAM–TAAM and HAR–TAAM in *NoSpherA2*

Hybrid-mode refinements were performed in two ways. In the first case, IAM–TAAM hybrid refinement was used for the coordinated metal–organic complex, where the metal atoms were refined spherically using IAM and organic parts were treated aspherically using TAAM. In the second case of hybrid HAR–TAAM refinement, the metal atoms were treated using HAR and organic molecules were treated using TAAM. The metal and organic parts were parametrized either with formal charge assigned to them or as neutral moieties. A level of theory of B3LYP/6-31G(*d*,*p*) was used for HAR in hybrid refinement. The hybrid refinements were performed like the disorder case, where separate .tsc files were generated for each part and combined before the final refinement. Other refinement settings for HAR are described in the previous section. More details can be found in the supporting information.

## 3. Results

### 3.1. Comparison of IAM refinements obtained from *SHELXL* as originally reported and from *olex2.refine* used in this study

The reported structures (Fig. 1 ▸) were originally refined with *SHELXL* (Sheldrick, 2015 ▸). In this paper, we have used *olex2.refine* (Dolomanov *et al.*, 2009 ▸) for the IAM refinement. The initial models obtained from IAM using *olex2.refine* were used as a basis for further refinements using TAAM or HAR. Although both *SHELXL* and *olex2.refine* are based on least-squares refinements and the same IAM scattering model, there are subtle differences in how the reliability factors (*R*1) and residual densities are calculated by these two programs. While these differences were not so apparent for good-quality data when refined with *SHELXL* or *olex.2refine*, the differences became more prominent when the data were of poor quality or the data were collected at high temperature. Here we have compared the refinement statistics of structures **1**–**11** which are purely organic systems refined at atomic resolutions (*d*_min_ in the range of 0.65–0.89 Å). Structure **12**, a network structure consisting of an aqueous sodium salt of methyl pyridone, will be discussed separately.

In structures **1** and **2** the data sets were merged and are of good quality (Fig. S1); there were no differences in reliability factors and residual densities obtained from *SHELXL* and *olex2.refine* (Table 1 ▸). For structures **3**–**6**, the data quality was poor with low *I*/σ, especially in the high-resolution region (Table 1 ▸ and Fig. S1), and the model did not fit well to the data, as indicated by a very high *R*1 for the high-resolution region [2θ > 35° (sin θ/λ > 0.42 Å^−1^); θ is the incident angle, *I*/σ the signal-to-noise ratio and λ the incident beam wavelength]. The differences between refinements in *SHELXL* and *olex2.refine* were visible in the refinement statistics, especially in residual densities (Table 1 ▸). For some of the structures **3**–**6**, data collected at low temperature (**4**_100 K_ and **6**_100 K_) were of better quality than those collected at ambient temperatures (**4**_300 K_ and **6**_300 K_) (Fig. S1). The differences in refinement statistics resulting from the usage of different software were also minimal in structures **4**_100 K_ and **6**_100 K_. The reported structure **7** showed disorder in the terminal alkenyl chain which was not modelled, as can be seen from the large displacement ellipsoids and residual densities [Fig. 1 ▸ and Table 1 ▸ (**7**)]. The *R*1 and residuals were reduced after modelling the disorder in *olex2.refine*. The major and minor conformers have occupancies of 0.65 and 0.35, respectively. In the case of COFs **8** and **9**, again the data quality was poor due to trapped solvent which could not be modelled properly and was masked in the IAM refinement (Fig. S1). Differences in *R*1 and the residual densities in the case of **8** and **9** obtained from *SHELXL* and *olex2.refine* were apparent (Table 1 ▸). For structure **10**, the data quality was good and there was not much difference between *SHELXL* and *olex2.refine* results. However, a high residual density was observed in structure **10** (Table 1 ▸). In the case of the twinned structure **11**, the data quality was poor at high resolution [2θ > 35° (sin θ/λ > 0.42 Å^−1^)] and slightly higher residuals were observed in *olex2.refine* compared with the reported values (Table 1 ▸ and Fig. S1).

### 3.2. Comparison of IAM and TAAM refinements

The improvement in refinement statistics from TAAM refinement compared with IAM has been discussed on numerous occasions (Bąk *et al.*, 2011 ▸; Sanjuan-Szklarz *et al.*, 2016 ▸; Jha *et al.*, 2020 ▸). TAAM refinement has been generalized on a set of small organic molecules using the MATTS2021 databank to achieve a better description of atom positions, hydrogen-bond distances and anisotropic displacement parameters (Jha *et al.*, 2020 ▸). However, it was not possible to perform such a comparison on structures involving disorder, twinned structures, MOFs *etc*. Integration of TAAM into *NoSpherA2* now enables refinement and comparison of the improvements in these classes of crystal structures.

In the following we will compare the results obtained from IAM and TAAM refinements for structures **1**–**11**. Structures **1** and **2** show an improvement of ∼1 percentage point in *R*1 and >50% of the electron-density residuals after TAAM refinements, compared with IAM. This is in good agreement with our previous study (Jha *et al.*, 2020 ▸). For the multi-component structures **3**–**6**, only structures **4**_100 K_ and **6**_100 K_ showed considerable improvement in *R*1 and residual densities upon the description of the aspherical density. As mentioned earlier, the data quality of structures **4**_100 K_ and **6**_100 K_ was much better than that for the other multi-component structures **3**–**6** (Fig. S1). For **3**, **4**_300 K_, **5** and **6**_300 K_ the TAAM refinement showed a small improvement in *R*1; however, the residual densities in these structures were found to be even higher than for the IAM refinement. We also compared the residual electron-density map and fractal dimension plot for structure **3**_100 K_ as one of the representatives of structures **3**–**6** (Figs. S2 and S3). A parabolic shape of a fractal dimension plot is an indicator of Gaussian noise distribution on a residual map and data are considered devoid of any systematic error (Meindl & Henn, 2008 ▸). The residual electron-density map from TAAM was found to be more populated and less featureless compared with that from IAM; also the fractal dimension plot showed behaviour that deviated more from ideal Gaussian noise in TAAM than in IAM. These observations again highlight the poor quality of the data and the sensitivity of TAAM refinement in detecting problematic data (Jha *et al.*, 2020 ▸).

**Figure 2:**
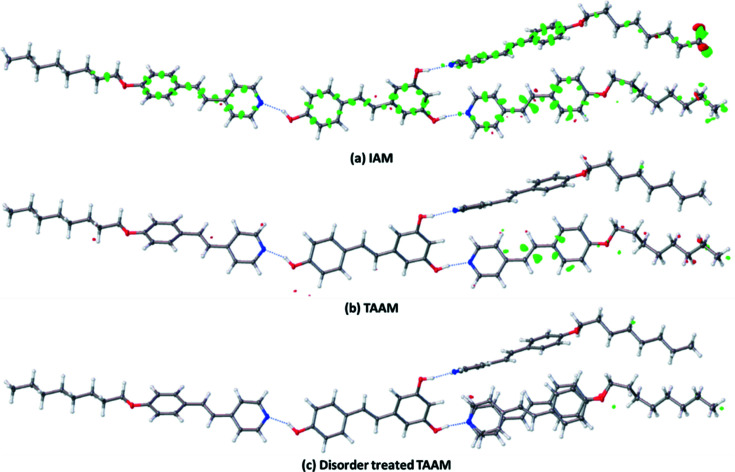
Comparison of residual density maps obtained for structure **10** from IAM (*a*) and TAAM [(*b*), (*c*)] refinements. The presence of disorder is easily visible in the residual density obtained from the TAAM refinement (*b*), but was not distinguishable from the unmodelled bonding density in IAM (*a*). The disorder-treated TAAM (*c*) clearly shows a featureless residual density map. The contour level was set to 0.35 e Å^−3^.

In the data set of structure **7**, the data quality was good (Fig. S1), but the initial TAAM refinement led to higher residual densities than the corresponding IAM refinement, clearly supporting the observation that there is unmodelled disorder in the structure. After appropriate modelling of the disorder, TAAM refinement showed further improvements in *R*1, residual densities and fractal plots [Table 1 ▸ and Fig. S7(*a*)]. The fractal dimension plot of the reported IAM structure of **7** with disorder unmodelled deviates greatly from the parabolic shape and indicates the biases in the model [Fig. S7(*a*)]. The fractal dimension plots after proper disorder treatment are parabolic for IAM and TAAM refinement, but the plot is much steeper for TAAM refinement [Fig. S7(*a*)]. COFs **8** and **9** both had disordered solvents, which could not be modelled and were masked. These refinements of structures with masked solvent disorder using TAAM are only possible in *NoSpherA2*. Structures **8** and **9** also showed issues with data quality, which is reflected in the TAAM refinements by a high *R*1 and residual densities (Table 1 ▸). In structure **8**, the data at high resolution were found to be mostly contributing to noise (Fig. S1). The residual electron-density map and fractal dimension plot indicated a small improvement in TAAM compared with IAM for structure **8** (Figs. S4 and S5). Structure **10** shows higher residual density after TAAM refinements than after IAM, although the data quality for structure **10** appears to be without problems (Fig. S1). TAAM refinement results in an almost featureless residual density map except around the C=C bond and ring of one component in the structure [Fig. 2 ▸(*b*)], which clearly shows the presence of disorder that was not visible in the IAM results [Fig. 2 ▸(*a*)]. The TAAM refinement, which included proper modelling of the disorder, further improved the structure model (*R*1_gt_ = 3.31% compared with 3.71% for TAAM with disorder not modelled) and gives a featureless residual density map [peak/hole = 0.36/−0.35 (e Å^−3^) compared with 0.72/−0.46 (e Å^−3^) for TAAM with disorder not modelled] [Table 1 ▸ and Fig. 2 ▸(*c*)]. The minor conformer refined to an occupancy of 0.0430 (16) for TAAM and to 0.048 (2) for IAM. Structure **11** is twinned; the data quality was poor and we observed a higher residual density in TAAM compared with IAM. The comparison of the C—C bond-length precision obtained from IAM and TAAM on structures **1**–**11** showed significant changes; the TAAM refinement showed better precision compared with IAM in all cases except in **8** and **9** (Table S1 and Fig. S6). In the case of **8** and **9** there was unmodelled solvent disorder which was left out of the refinement and, since TAAM is sensitive to such unmodelled atoms, this may lead to higher values of fitting statistics and lower precision compared with IAM.

We can conclude that, when employing TAAM refinements, any unmodelled features, like disorder or data quality issues, are more clearly exposed. Similarly to our earlier observations (Jha *et al.*, 2020 ▸) and despite the issues of data quality, it was always possible to observe significant improvements in the geometry, displacement parameters and hydrogen-bond lengths in TAAM compared with IAM. The detailed comparisons of these individual parameters will be exemplified on a selected model structure later in the text.

### 3.3. Comparison of IAM and hybrid refinement on selected model structures

Hybrid refinements were performed for structure **12** using IAM–TAAM and HAR–TAAM and compared with the IAM results. The hybrid refinements were performed with and without assignment of formal charges to the sodium atom and hy­droxymethyl­pyridone, respectively.

**Figure 3:**
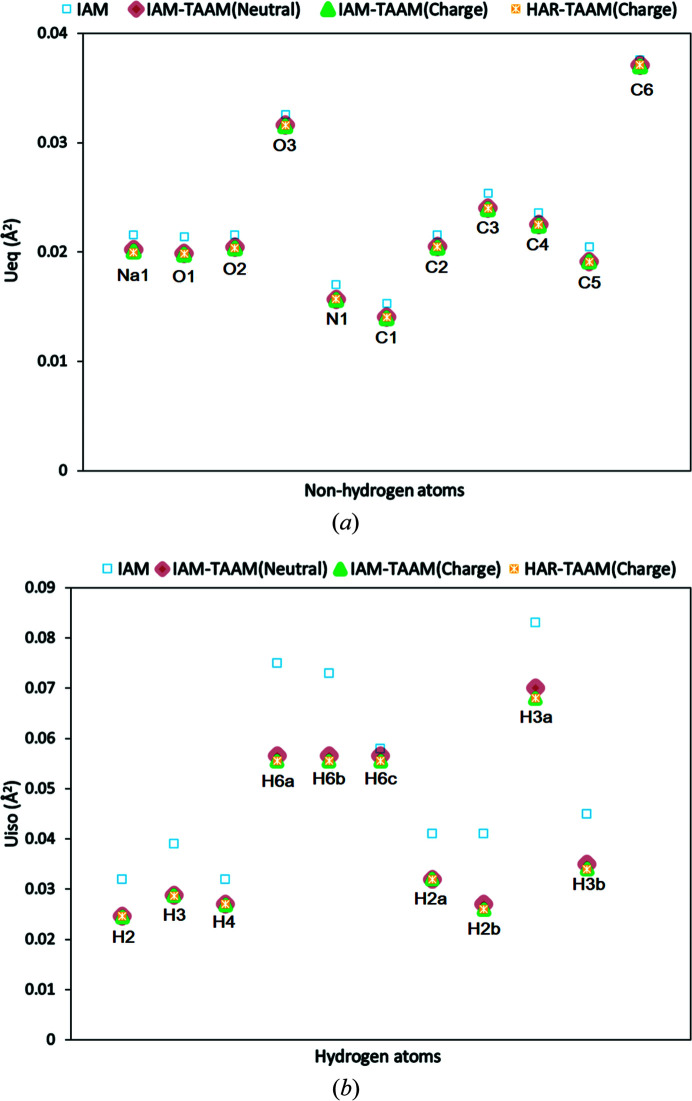
Comparison of (*a*) *U*_eq_ (Å^2^) for non-hydrogen atoms and (*b*) *U*_iso_ (Å^2^) for hydrogen atoms obtained for structure **12** from IAM and hybrid refinements.

The improvements in *R*1 and electron-density residuals from hybrid refinement compared with the IAM results were found to be similar to those of pure HAR (Woińska *et al.*, 2016 ▸) and TAAM refinements (Jha *et al.*, 2020 ▸) in structures with good data quality. For the hybrid IAM–TAAM refinement of structure **12**, without assigning any formal charges, an improvement of ∼0.7 percentage points in *R*1 and ∼0.13 e Å^−3^ in residual electron density was found (Table 2 ▸). We observed no significant changes in refinement statistics between the IAM–TAAM neutral and IAM–TAAM and IAM–HAR with formal charge hybrid models (Table 2 ▸). The comparison of displacement parameters *U*_eq_ (non-hydrogen atoms) and *U*_iso_ (hydrogen atoms) between IAM and hybrid refinements showed lower values for the hybrid refinements (Fig. 3 ▸). However, there were no significant differences found in *U*_eq_ (non-hydrogen atoms) and *U*_iso_ (hydrogen atoms) among the different types of hybrid refinements (Fig. 3 ▸). The *U*_iso_ for hydrogen atoms in the hybrid refinements showed the opposite trend to those for pure TAAM refinement, where the *U*_iso_ values for hydrogen atoms were found to be higher than those from IAM (Jha *et al.*, 2020 ▸).

**Figure 4:**
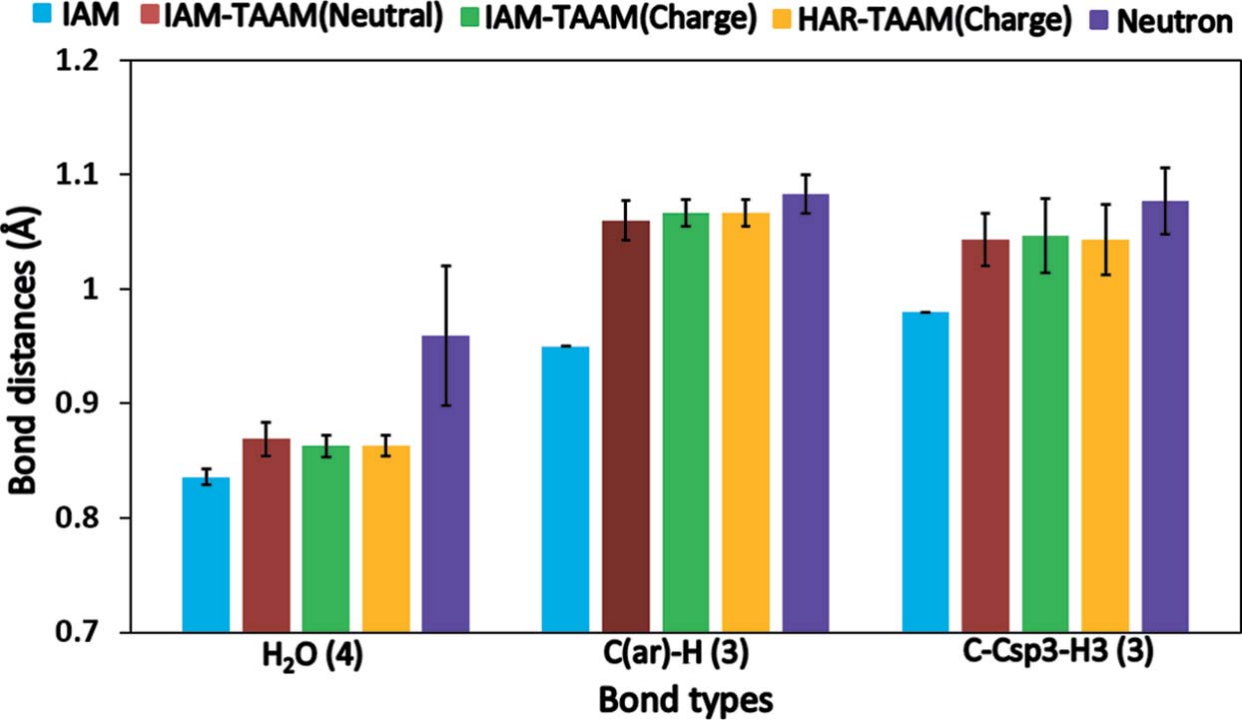
Comparison of the *X*—H average bond lengths of various bond types [H_2_O, C(ar)—H, C—C*sp* ^3^—H_3_] for structure **12** with neutron bond lengths as defined previously (Allen & Bruno, 2010 ▸). The O—H bonds in water molecules are compared with the corresponding neutron bond lengths taken from Woińska *et al.* (2016 ▸). The numbers in parentheses in the bond type labels indicate the number of occurrences.

There were three types of bonds involving hydrogen atoms in structure **12**, namely the hydrogen atom attached to (*a*) oxygen in water (H_2_O), (*b*) carbon in the aromatic ring [C(ar)—H] and (*c*) carbon in a terminal methyl group (C—C*sp* ^3^—H_3_). A comparison of the *X*—H bond lengths shows a significant improvement in hybrid refinements compared with the IAM refinement in all three cases, with the data approaching corresponding neutron bond lengths (Allen & Bruno, 2010 ▸). There is no significant difference in the *X*—H bond lengths between different hybrid-mode refinements. The improvement in bond lengths compared with IAM was approximately 4, 12 and 7% for H_2_O, C(ar)—H and C—C*sp* ^3^—H_3_ bond lengths, respectively (Fig. 4 ▸).

### 3.4. Comparison of accuracy and computational costs in IAM, HAR and TAAM refinements on selected model structures

We used structures **7** and **3**_100 K_ for the comparison of IAM, HAR and TAAM refinements. Structure **7** has strong and high-resolution data. Additionally, it shows disorder. Structure **3**_100 K_ has comparatively weak data. The IAM refinement (*SHELXL* or *olex2.refine*) on both structures converges in less than 10 s (Tables 2 ▸ and S2). However, *R*1 and the residual densities were higher in the IAM results compared with the aspherical refinements in the case of structure **7**, while for structure **3**_100 K_ the residuals were lower.

We used B3LYP along with different basis sets for the wavefunction calculation for HAR for structures **7** and **3**_100 K_. *R*1 and the residual densities improved with higher basis sets; however, the time taken for the refinement increased exponentially with the complexity of the basis sets in HAR. It took around 7–8 min for a small basis set (3-21G) to run one cycle of HAR, which consists of wavefunction calculation, scattering factor calculation from the wavefunction and finally the least-squares refinement. To achieve convergence the whole process was repeated iteratively, and it took 24 min for **3**_100 K_ (Table S2) and 39 min for structure **7** (Table 3 ▸). Moderate basis sets such as 6-31G(*d*,*p*) took around 1–1.5 h to achieve convergence in HAR. It took around 5–6.5 h for a higher basis set such as Def2-TZVP in HAR to achieve convergence (Tables 3 ▸ and S2). The improvement in *R*1_gt_ and the residuals going from 3-21G to Def2-TZVP was around 0.13 percentage points and 0.05/−0.06 e Å^−3^, respectively, for **3**_100 K_ (Table S2). For structure **7** *R*1 improves by ∼0.15 percentage points and there was no significant difference in residuals (Table 3 ▸), while there was a tenfold increase in time in both cases.

The refinement statistics for TAAM were found to be comparable to those obtained by HAR. A close resemblance was found with HAR using 6-31G(*d*,*p*) (Tables 3 ▸ and S2). The B3LYP functional and 6-31G(*d*,*p*) basis set were also used for the calculation of wavefunctions for the creation of the MATTS2021 databank (Jha *et al.*, 2022 ▸) used in this study. With a larger basis set (Def2-TZVP) in HAR *R*1 decreases, but the highest and lowest residuals remain almost the same. One cycle of TAAM refinement, consisting of databank transfer, scattering factor calculation and finally the least-squares refinement, took around 20 s, which is on the same order as that of the IAM refinement. To achieve convergence iteratively the TAAM refinement took 2 and 5 min for structure **3**_100 K_ (Table S2) and **7** (Table 3 ▸), respectively.

We also compared the fractal dimension plots from the different models for structure **7**. The comparison of the fractal dimension plots between TAAM and HAR with different basis sets for disorder-treated structure **7** [Fig. S7(*b*)] shows a parabolic shape with minimal variations among the different models [Fig. S7(*b*)].

**Figure 5:**
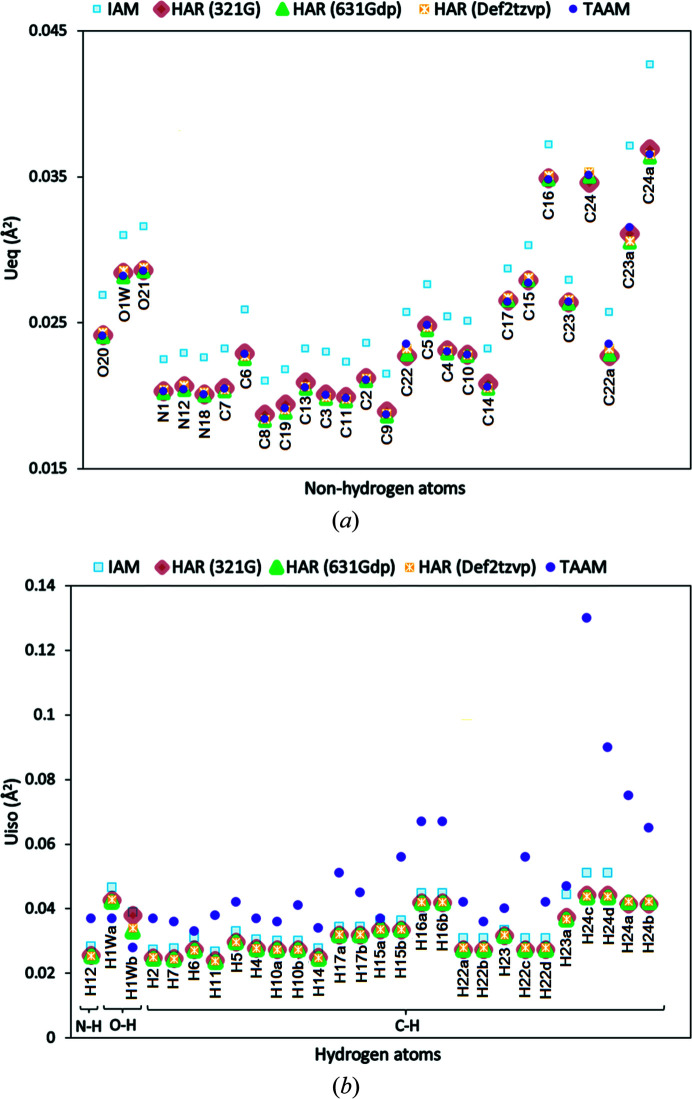
Comparison of displacement parameters of structure **7** after IAM, HAR and TAAM refinements: (*a*) *U*_eq_ (Å^2^) for non-hydrogen atoms, (*b*) *U*_iso_ (Å^2^) for hydrogen atoms.

Additionally, a comparison of the atomic displacement parameters between IAM, HAR and TAAM for non-hydrogen (anisotropic, *U* ^*ij*^) and hydrogen atoms (isotropic, *U*_iso_) was performed. The *U* ^*ij*^ values of non-hydrogen atoms were compared by focusing on their equivalent isotropic displacement parameters (*U*_eq_) (Fischer & Tillmanns, 1988 ▸). Aspherical refinements – both HAR and TAAM – showed a 10–11% decrease in all atomic displacement parameters compared with the corresponding atomic displacement parameter from IAM [Figs. 5 ▸(*a*) and S8(*a*)] for non-hydrogen atoms. There was no significant difference between the average *U*_eq_ of non-hydrogen atoms obtained from HAR using different basis sets and TAAM. Significant differences appeared in *U*_iso_ values for hydrogen atoms when using HAR or TAAM. In the case of structure **7**, the average *U*_iso_ from HAR was ∼9% smaller compared with the IAM results, while the average *U*_iso_ using TAAM was higher (∼28%) compared with the IAM results [Fig. 5 ▸(*a*)]. The overall difference between HAR and TAAM average *U*_iso_ values for hydrogen atoms in **7** was ∼34%. In structure **7**, the nitro­gen- and carbon-bound hydrogen atoms showed higher *U*_iso_ values and the oxygen-bound hydrogen atoms lower *U*_iso_ values in the TAAM refinement compared with IAM and HAR [Fig. 5 ▸(*b*)]. In **3**_100 K_, the *U*_iso_ values for C—H atoms from HAR and TAAM showed a different trend, with higher values compared with those from IAM [Fig. S8(*b*)]. The *U*_iso_ values for O—H and N—H hydrogen atoms from HAR and TAAM were slightly smaller than those from IAM. The *U*_iso_ of hydrogen atoms obtained from HAR using different basis sets showed very similar values. The average TAAM *U*_iso_ for C—H hydrogen atoms increased by approximately 34%, while after HAR the corresponding difference was only around 16% compared with the IAM results [Fig. S8(*b*)].

**Figure 6:**
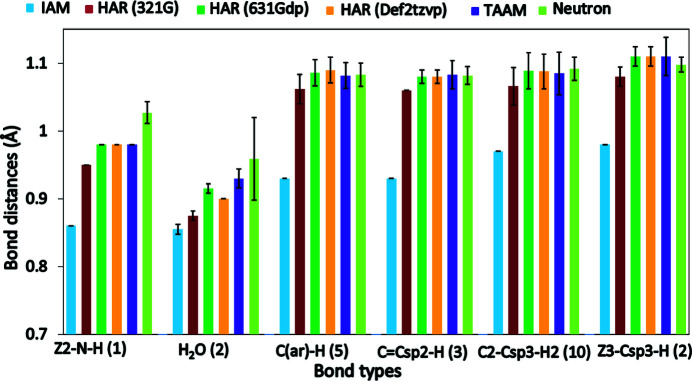
Comparison of *X*—H average bond lengths of various bond types [Z2—N—H, H_2_O, C(ar)—G, C=C*sp* ^2^—H, C2—C*sp* ^3^—H2, Z3—C*sp* ^3^—H] for structure **7** with neutron bond lengths as defined previously (Allen & Bruno, 2010 ▸). The O—H bonds in water molecules are compared with the corresponding neutron bond lengths taken from Woińska *et al.* (2016 ▸). The numbers in parentheses in the bond type labels indicate the number of occurrences.

A comparison of the *X*—H bond lengths reveals that the IAM bond lengths were the shortest in both structures **7** (Fig. 6 ▸) and **3**_100 K_ (Fig. S9) compared with HAR, TAAM and neutron bond lengths, which is consistent with earlier findings (Woińska *et al.*, 2016 ▸; Jha *et al.*, 2020 ▸). The bond lengths obtained from TAAM refinement were comparable to those obtained by HAR in both structures. The TAAM bond lengths were moderately longer compared with those from HAR employing the 3-21G basis sets and slightly shorter in the case of the higher basis sets, 6-31G(*d*,*p*) and Def2-TZVP (Figs. 6 ▸ and S9). The biggest differences appeared in the case of water molecules in both structures **7** and **3**_100 K_. In structure **7** with good data quality, the O—H bond lengths of the water molecule from both HAR and TAAM were comparable to neutron bond lengths and much longer than those from IAM (Fig. 6 ▸). In the case of structure **3**_100 K_, the O—H bond lengths were overestimated in all aspherical refinements and were the longest for TAAM (Fig. S9). This confirms that, irrespective of the data quality, there is a significant improvement in structure models for both bond lengths and displacement parameters from any aspherical refinement compared with the spherical IAM refinement.

## 4. Discussion

In the case of good-data-quality structures, TAAM refinement showed considerable improvement in refinement statistics such as *R*1 and electron-density residuals compared with IAM. The *R*1 in good-quality structures was reduced by ∼1 percentage points and electron-density residuals by ∼50%. The achievement of better fitting statistics confirms that aspherical refinement is more accurate than IAM and allows us to describe better the physical reality of the sample. While there is always a risk of overfitting, here the risk seems to be the same for TAAM (and HAR) as for IAM, since the same number of parameters is refined. Moreover, in the case of TAAM, like for IAM, the values of electron-density parameters needed to build the model are obtained beforehand. To a large extent, they do not depend on the current geometry of the refined structure, and thus there is no risk of artificially freezing the structure at the false minimum.

With the more credible models, there is less room for error or misinterpretation of the data. By removing the systematic error present in IAM resulting from a lack of modelling of bonding features, other as-yet unmodelled features are more visible. Aspherical refinements may help us to understand the origin of the remaining errors, and sometimes allow for error elimination by building a correct model taking into account their source. For example, disordered regions of a molecule are easier to detect and interpret. The sensitivity of TAAM refinement towards any problems in the measured data or unmodelled features in refined structures was shown by higher *R*1 values and electron-density residuals. In some cases where the data or model quality was very poor, the electron-density residuals obtained from TAAM were found to be even higher than those from IAM. In the case of the disordered structures **7** and **10**, where the data quality was good, after proper modelling of the disorder, TAAM refinement showed similar improvements in *R*1 (∼1%) and residuals (∼50%). These observations are consistent with earlier findings (Jha *et al.*, 2020 ▸).

A comparison of IAM, HAR and TAAM refinement on one of the model structures showed that the time taken by TAAM is of a similar order of magnitude to the time taken for IAM, while HAR takes 50 times longer with a small basis set (3-21G). The time taken by HAR refinements increases exponentially with larger basis sets: refinement takes several minutes to hours, whereas with IAM it takes less than 10 s. In the case of TAAM refinements, it took less than 20 s for one cycle of refinement, while for iterative refinements it took 2–5 min to achieve convergence.

The refinement statistics such as *R*1 and residual electron density from TAAM were comparable to those from HAR, with slightly lower values in favour of HAR with a very high basis set (Def2-TZVP). The displacement parameters *U*_eq_ for non-hydrogen atoms in HAR and TAAM were smaller than those from IAM. For hydrogen atoms, the trend in *U*_iso_ values depended upon the data quality and the atom type to which the hydrogen atoms were attached. In the case of strong good-quality data, the *U*_iso_ values for hydrogen atoms from HAR were smaller than those from IAM, and for TAAM they were significantly bigger than those from IAM, except in the case of O—H hydrogen atoms where the TAAM *U*_iso_ values were smaller than those of HAR. In the case of weak poor-quality data the *U*_iso_ values for C—H hydrogen atoms were larger in both HAR and TAAM compared with IAM. Hydrogen atoms attached to the more electronegative oxygen and nitro­gen show smaller *U*_iso_ values from HAR and TAAM in comparison with IAM. The *X*—H bond lengths from HAR and TAAM showed considerable improvement compared with the IAM results and approached neutron bond lengths. The bond lengths obtained from TAAM refinements were comparable to those obtained by HAR, although on the slightly smaller side in the case of a higher basis set.

We have introduced the hybrid IAM–TAAM and HAR–TAAM refinements for coordination compounds and network salt-containing metal ions. In the hybrid IAM–HAR–TAAM, various chemical moieties can be parametrized using different scattering models to speed up the procedure or to overcome the lack of proper parametrization in TAAM or HAR. The improvements in the refinement statistics such as *R*1 and residual electron density when using the hybrid model were similar to those achieved by full HAR or TAAM in comparison with the IAM refinement. The non-hydrogen and hydrogen atomic displacement parameters were found to be lower in hybrid refinements than in IAM refinement. The lower value of hydrogen atomic displacement parameters obtained from hybrid refinement compared with IAM are in contrast to the pure TAAM refinement where the atomic displacement parameters for hydrogen atoms were higher (Jha *et al.*, 2020 ▸). However, there were no significant differences among atomic displacement parameters, irrespective of the different hybrid methods used for both non-hydrogen and hydrogen atoms. There was a significant improvement in bond lengths to hydrogen atoms from hybrid refinements compared with the IAM results, and the bond lengths approached neutron values (Allen & Bruno, 2010 ▸). Hybrid refinements were much quicker than the conventional HAR approach.

## 5. Conclusions

TAAM refinements in *NoSpherA2* on a diverse set of structures, containing organic small molecules, co-crystals, disorder, twinning, and network and polymeric structures such as COFs and salts containing metal ions, were performed successfully and compared with IAM refinements.

There were clear benefits of using aspherical models for crystal structure refinement on standard X-ray diffraction data, irrespective of the quality of the data and resolution.

With good-quality X-ray data and a correct atomic model, the increase in reliability of the structural model achieved from TAAM refinement was manifested by a reduction of *R*1 by ∼1 percentage points, lowering of the electron-density residual by ∼50%, improved accuracy of the *X*—H bond lengths usually by ∼0.2 Å and improved C—C bond precision by 0.0004 Å. Clearly better electron-density models allowed us to obtain more accurate and more precise geometrical parameters than were accessible from IAM refinements.

Whenever the above-mentioned indicators did not improve, this pointed to poor quality of the experimental data or the presence of systematic errors in the data, the source of which was not properly accounted for by the models applied during data reduction or refinement. Thanks to aspherical refinement, the presence of disorder was easily detected and appropriate modelling of it was confirmed by improvements in fitting statistics.

Aspherical refinement on X-ray data, compared with IAM, led to smaller values of displacement parameters *U*_eq_ in the case of most of the non-hydrogen atoms. For hydrogen atoms, the trends for *U*_iso_ parameters changed, depending on the type of atom to which the hydrogen atom was attached, and also the type of electron-density model (TAAM or various flavours of HAR) and the quality of the data. Understanding the reasons for that behaviour requires more study. Certainly, a model of atomic motion with a more physical framework would be beneficial here.

TAAM is available for most organic molecules, covering large areas of the Cambridge Structural Database, but its major limitation is the availability of atom types for much heavier elements. The main problem of HAR is the computation time. The hybrid IAM/TAAM/HAR method was introduced to benefit as much as possible from aspherical refinement. The comparable statistics among HAR, TAAM and hybrid-mode refinements give the crystallographer the choice of using various combinations of options under one software umbrella, without losing accuracy and precision, in a convenient and quick time frame.

Although one should proceed on a case-by-case basis, in general we can give the following recommendations:

(i) Never stop at the IAM refinement.

(ii) Perform TAAM refinement whenever possible, because it is fast and gives about the same structural parameters as HAR, including for hydrogen atoms.

(iii) Perform hybrid HAR/TAAM or pure HAR if TAAM is not possible.

(iv) Perform HAR if time is not an issue, or you want to account for fine features of electron density, like charge polarization by neighbouring molecules, relativistic effects *etc*.

(v) Perform aspherical refinement even with poor data or low resolution, because most often some improvements of the structural model are still achievable and often the source of problems in the data or modelling can be detected.

Although, for heavier elements, it seems that IAM is good enough to determine geometry, we still recommend carrying out aspherical refinement just to be sure that no hidden errors or unaccounted-for features are present in the data.

## Supplementary Material

Supporting figures and tables. DOI: 10.1107/S1600576722010883/te5103sup1.pdf

Cif files from all refinements. DOI: 10.1107/S1600576722010883/te5103sup2.bin

## Figures and Tables

**Table 1:** Comparison of refinements using different methods on X-ray diffraction data The subscript in Structure No. indicates the data collection temperature. dm, disorder modelled during the refinement. ND, not determined. gt, reflections fulfilling the following threshold: |*F*|^2^ > 2σ(|*F*|^2^). all, all the collected reflections.

		Reported IAM *SHELXL*			IAM *olex2.refine*			TAAM		
Structure No.	Resolution *d*_min_ (Å)	*R*1_gt_ (%)	*R*1_all_ (%)	Hole/peak (e Å^−3^)	*R*1_gt_ (%)	*R*1_all_ (%)	Hole/peak (e Å^−3^)	*R*1_gt_ (%)	*R*1_all_ (%)	Hole/peak (e Å^−3^)
**1**	0.84	3.33	3.64	0.18/−0.17	3.33	3.64	0.15/−0.16	2.27	2.52	0.09/−0.09
**2**	0.75	3.49	3.49	0.46/−0.26	3.48	3.48	0.47/−0.24	1.62	1.62	0.13/−0.14
**3**_100 K_	0.73	9.29	20.68	0.64/−0.51	9.25	20.82	0.96/−0.94	9.17	20.75	1.04/−1.03
**3**_300 K_	0.71	9.89	31.98	0.26/−0.32	9.92	31.99	0.98/−1.14	9.20	31.52	1.16/−1.11
**4**_100 K_	0.72	4.91	6.65	0.42/−0.27	4.92	6.65	0.46/−0.32	3.53	5.25	0.27/−0.26
**4**_300 K_	0.73	6.17	18.53	0.23/−0.19	6.18	18.61	0.49/−0.46	5.30	17.97	0.51/−0.52
**5**_100 K_	0.70	5.64	8.66	0.44/−0.36	5.64	8.65	0.50/−0.44	4.89	7.94	0.53/−0.39
**5**_300 K_	0.80	5.34	10.11	0.32/−0.16	5.34	10.10	0.35/−0.29	4.42	9.30	0.42/−0.26
**6**_100 K_	0.72	4.93	7.02	0.33/−0.20	4.94	7.02	0.39/−0.28	3.56	5.58	0.23/−0.24
**6**_300 K_	0.72	5.26	13.25	0.15/−0.16	5.18	13.23	0.27/−0.30	4.22	12.56	0.32/−0.27
**7**	0.80	5.67	5.75	1.19/−0.64	5.75	5.83	1.30/−0.62	5.04	5.10	1.41/−0.54
**7** ^dm^	0.80	ND	ND	ND	3.32	3.36	0.17/−0.19	2.32	2.37	0.11/−0.13
**8**	0.85	6.14	13.24	0.15/−0.13	6.18	13.03	0.35/−0.36	6.29	13.12	0.34/−0.29
**9**	0.89	7.61	8.64	0.26/−0.18	7.81	8.86	0.36/−0.22	7.83	8.91	0.37/−0.20
**10**	0.65	5.16	8.49	0.62/−0.33	5.17	8.50	0.69/−0.37	3.71	7.06	0.72/−0.46
**10** ^dm^	0.65	ND	ND	ND	ND	ND	ND	3.31	6.65	0.36/−0.35
**11**	0.75	4.32	8.37	0.20/−0.29	4.32	8.37	0.28/−0.48	3.92	8.09	0.34/−0.40

**Table 2:** Comparison of IAM and hybrid refinements for structure **12** All refinements were performed iteratively until convergence was achieved.

Model	*R*1_gt_ (%)	*R*1_all_ (%)	*wR*2_gt_(%)	*wR*2_all_ (%)	Peak/hole (e Å^−3^)
IAM	3.35	4.00	9.06	9.56	0.40/−0.20
IAM–TAAM (neutral)	2.62	3.27	6.59	6.94	0.26/−0.27
IAM–TAAM (charge)	2.64	3.29	6.71	7.05	0.27/−0.28
HAR–TAAM (charge)	2.64	3.29	6.69	7.05	0.27/−0.28

**Table 3:** Comparison of IAM, HAR and TAAM refinement statistics for crystal structure **7** HAR was performed using a level of theory of B3LYP with different basis sets, once for a single cycle and iteratively until convergence was achieved. TAAM refinement was also performed for a single cycle and iteratively.

Method	*R*1_gt_ (%)	*R*1_all_ (%)	*wR*2_gt_ (%)	*wR*2_all_ (%)	Peak/hole (e Å^−3^)	Time
*olex2.refine* IAM	3.32	3.36	8.77	8.33	0.17/−0.19	10 s
3-21G	2.72	2.76	7.46	7.53	0.11/−0.15	8 min
3-21G iterative	2.57	2.61	6.83	6.88	0.11/−0.15	39 min
						
6-31G(*d*,*p*)	2.61	2.65	7.17	7.24	0.14/−0.14	18 min
6-31G(*d*,*p*) iterative	2.41	2.45	6.39	6.43	0.12/−0.13	1 h 28 min
						
Def2-TZVP	2.65	2.69	7.24	7.31	0.14/−0.15	1 h 12 min
Def2-TZVP iterative	2.42	2.46	6.45	6.50	0.13/−0.14	6 h 26 min
						
TAAM	2.43	2.47	6.67	6.74	0.12/−0.13	20 s
TAAM iterative	2.32	2.37	6.18	6.23	0.11/−0.13	5 min
