# Supplementary material for: Aspherical atom refinements on X-ray data of diverse structures including disordered and covalent organic framework systems: a time–accuracy trade-off
Source: J Appl Crystallogr. 2023 Feb 1;56(Pt 1):116–27. doi: 10.1107/S1600576722010883 (PMC9901929; doi:10.1107/S1600576722010883)
Supplement: Supplementary file 1 [file j-56-00116-sup1.pdf]

JOURNAL OF
APPLIED
CRYSTALLOGRAPHY

Volume 56 (2023)

Supporting information for article:

Aspherical atom refinements on X-ray data of diverse structures including disordered and covalent organic framework systems: a time–accuracy trade-off

Kunal Kumar Jha, Florian Kleemiss, Michał Leszek Chodkiewicz and Paulina Maria Dominiak

Supplementary Material

Aspherical atom refinements on X-ray data of diverse structures including disorder and COF systems: A time-accuracy trade-off

Kunal Kumar Jha[1], Florian Kleemiss[2], Michał Leszek Chodkiewicz[1], Paulina Maria Dominiak[1]

1) Biological and Chemical Research Centre, Department of Chemistry, University of Warsaw, ul. Żwirki i Wigury 101, 02-089, Warszawa, Poland,

2) Universität Regensburg, Fakultät für Chemie und Pharmazie, Universitätsstr. 31, 93053 Regensburg, Germany.

Email of communicating author: pdomin@chem.uw.edu.pl

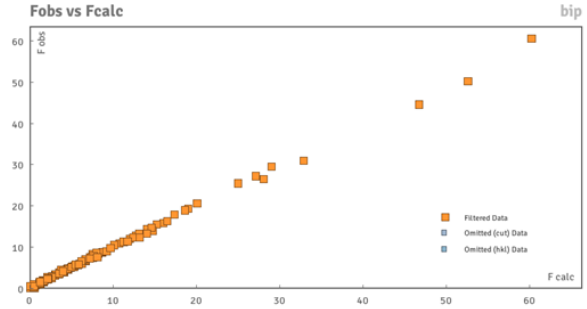

(a)

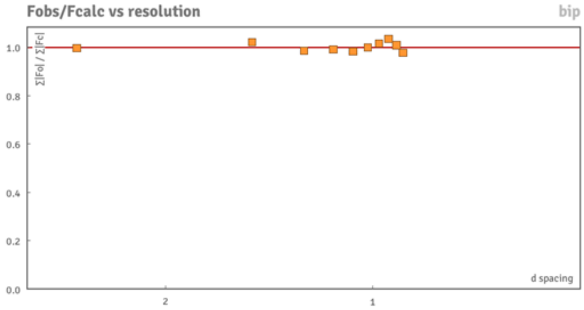

(b)

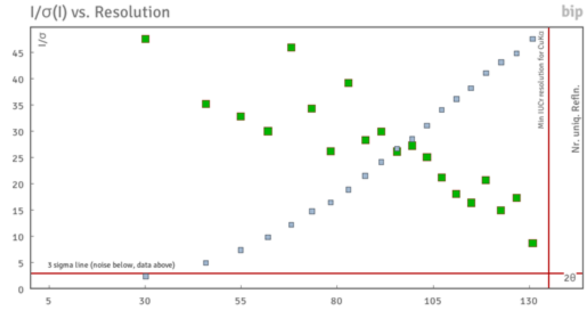

(c)

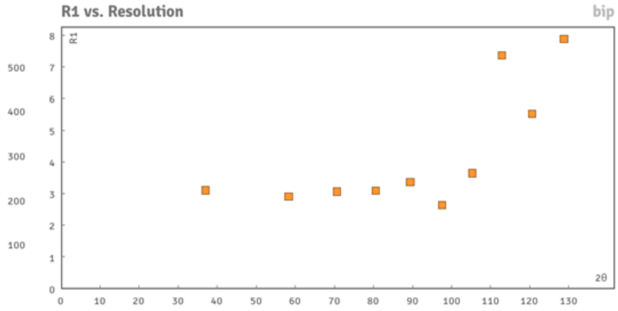

(d)

Structure **1**

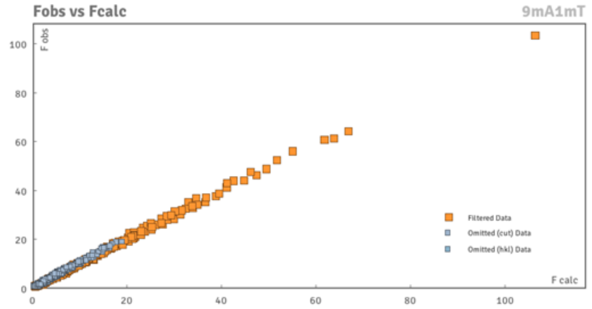

(a)

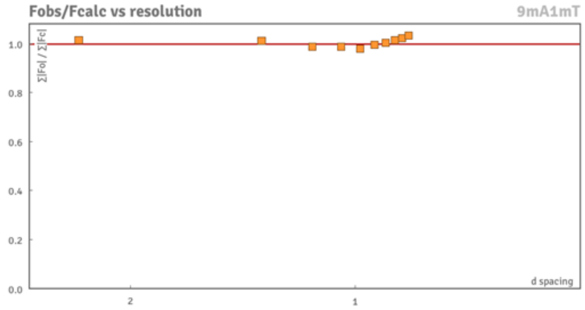

(b)

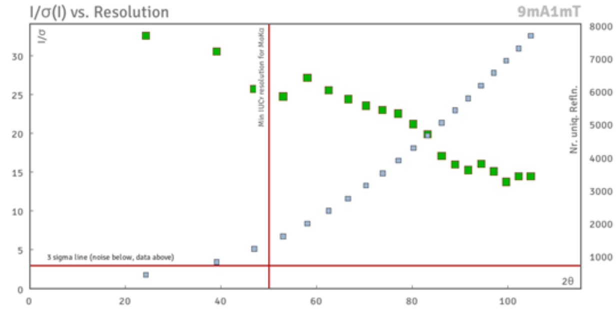

(c)

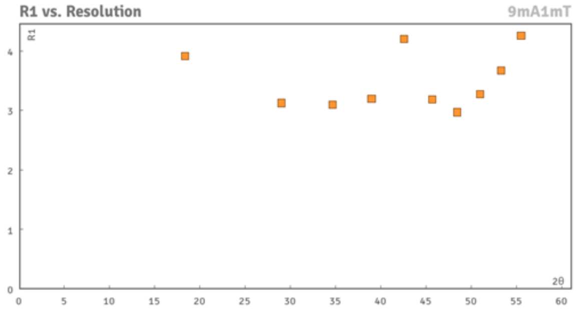

(d)

Structure **2**

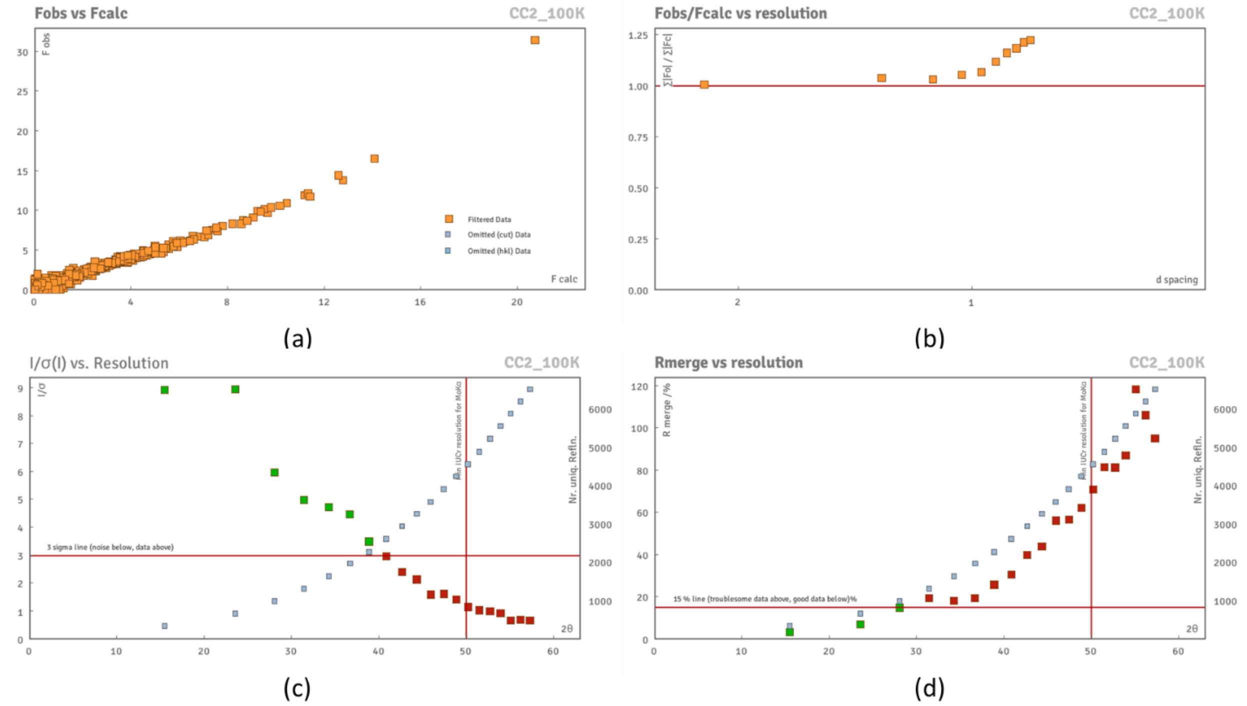

Structure **3**_100K

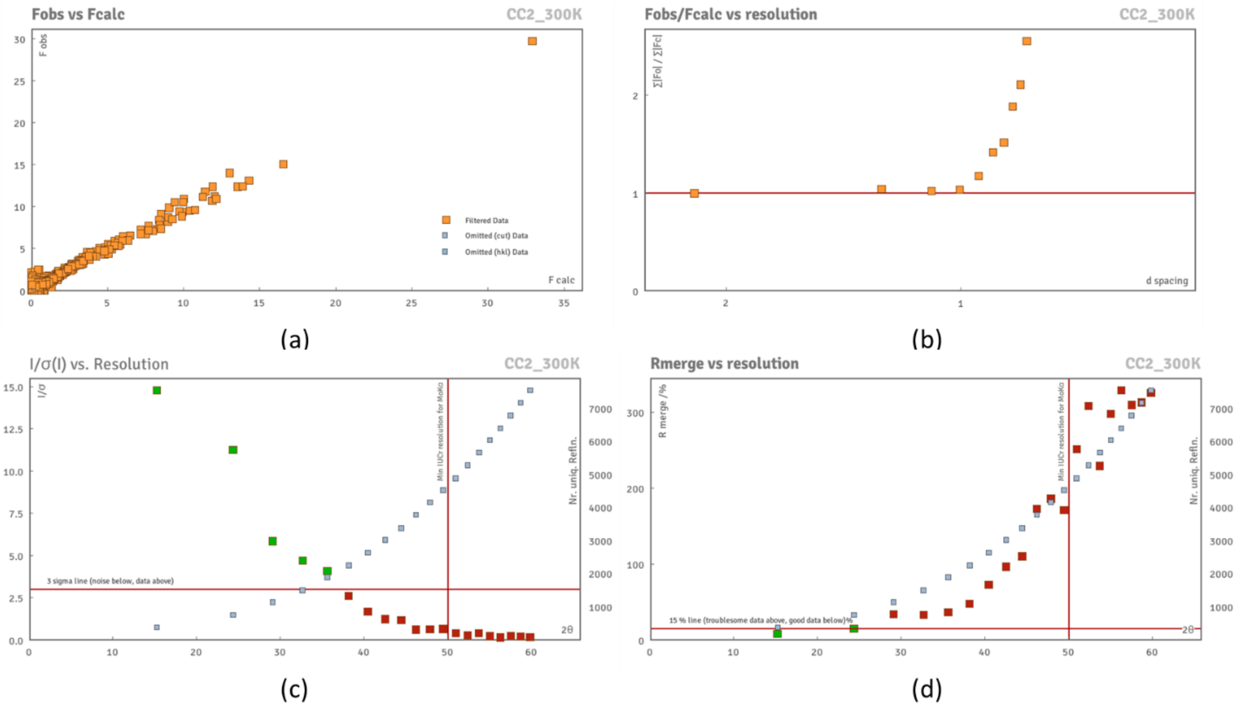

Structure **3**_300K

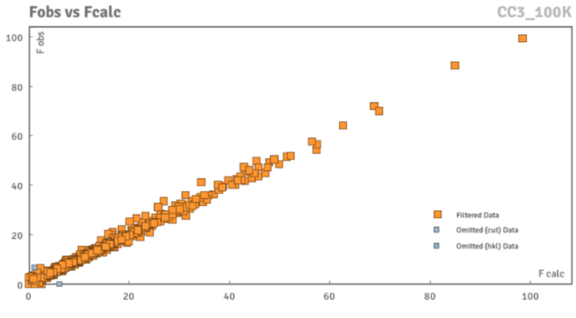

(a)

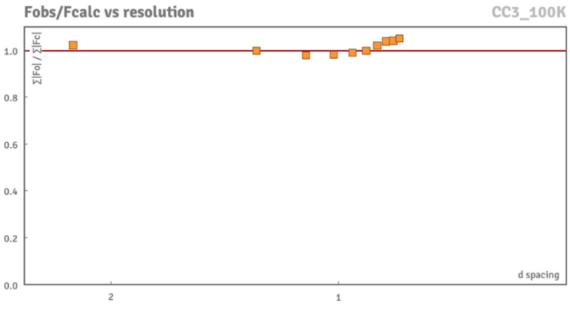

(b)

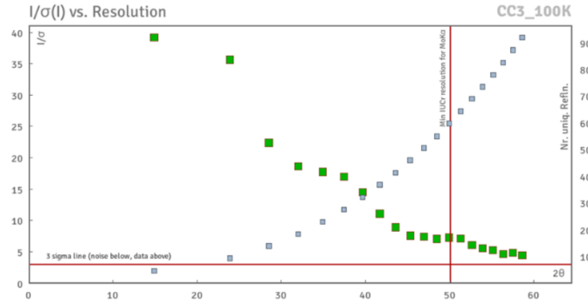

(c)

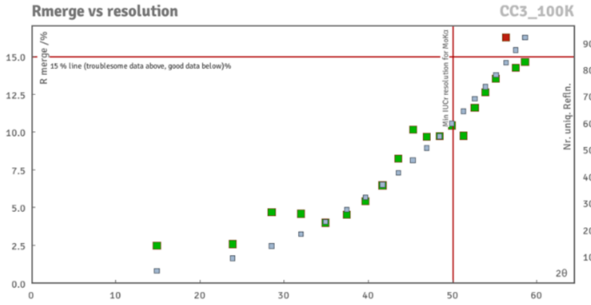

(d)

Structure **4**_100K

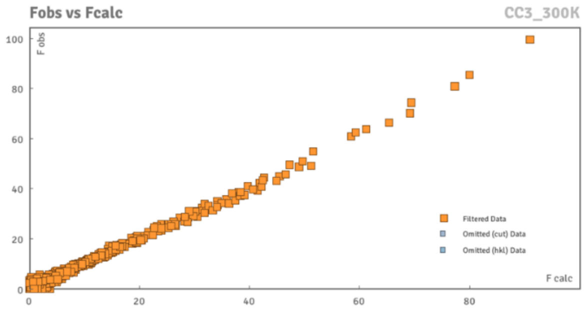

(a)

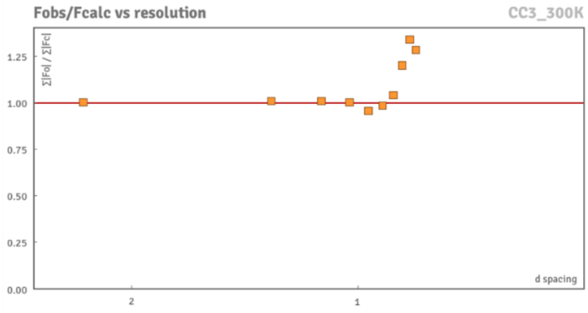

(b)

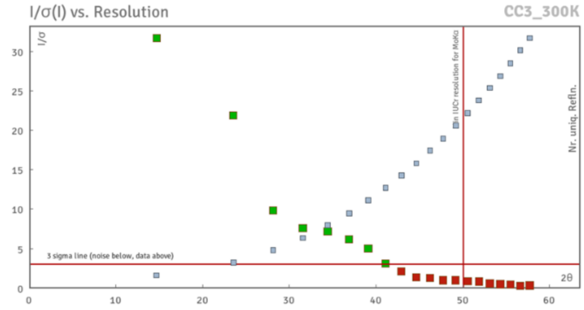

(c)

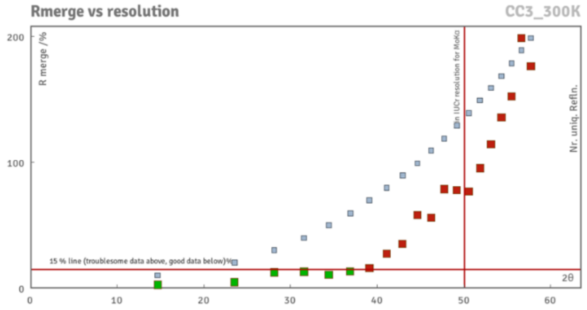

(d)

Structure **4**_300K

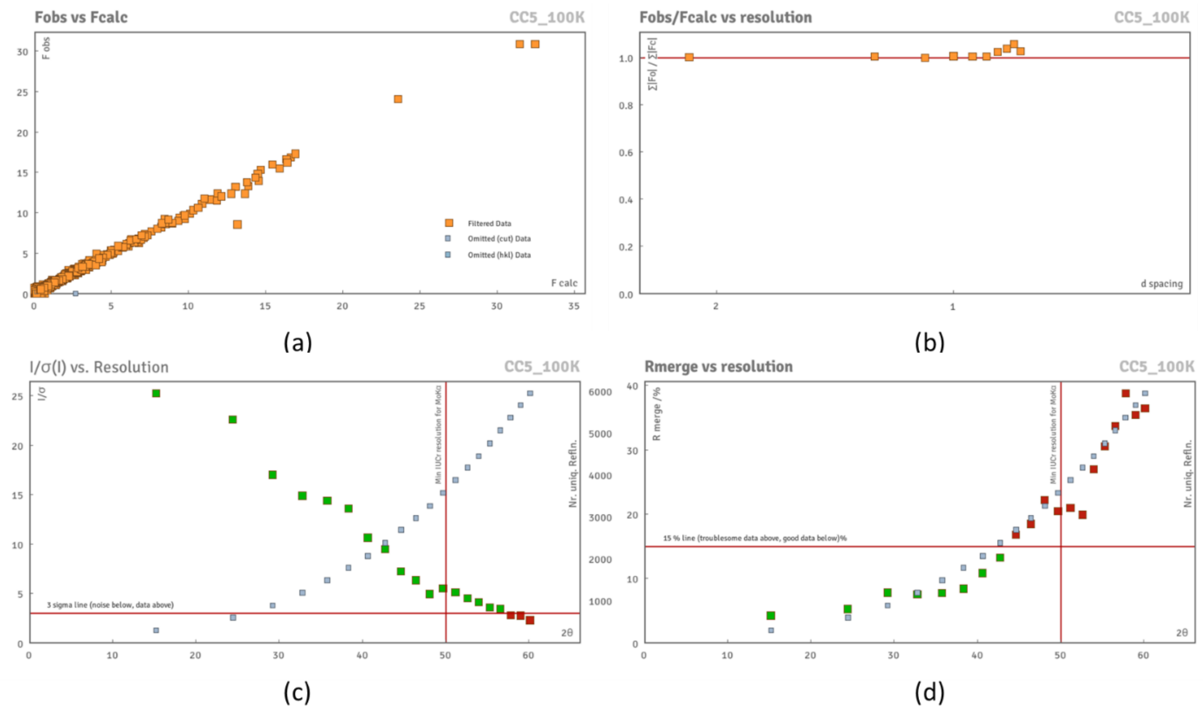

Structure **5**_100K

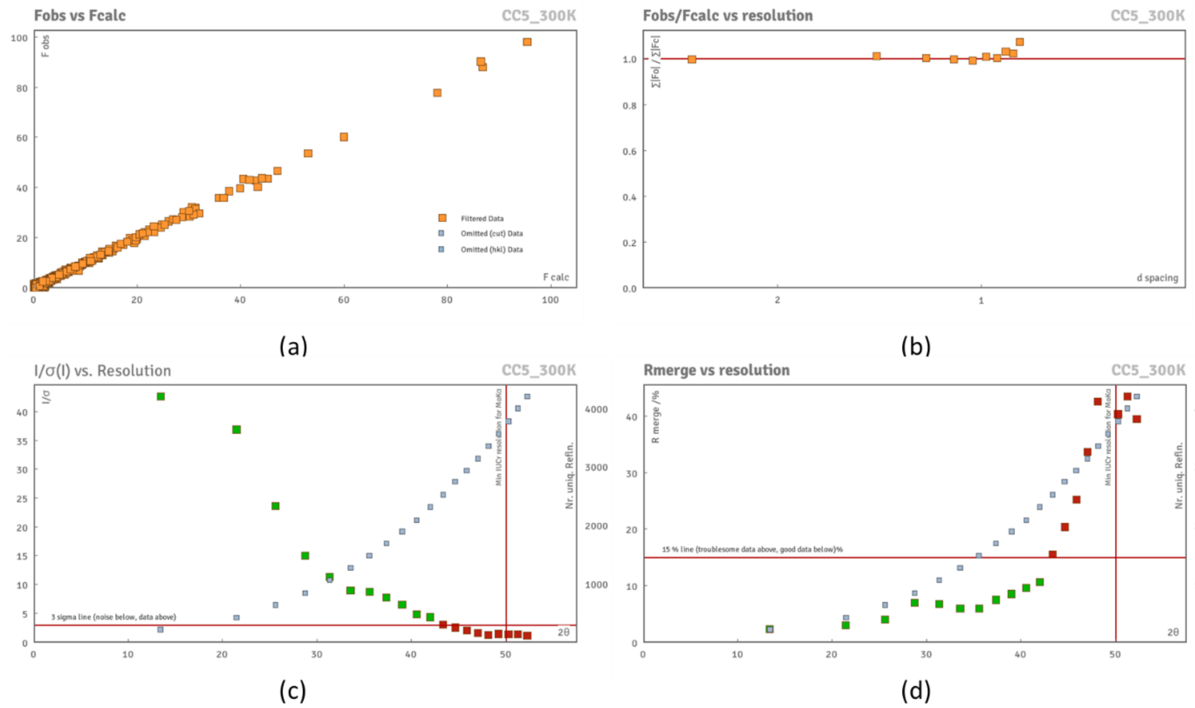

Structure **5**_300K

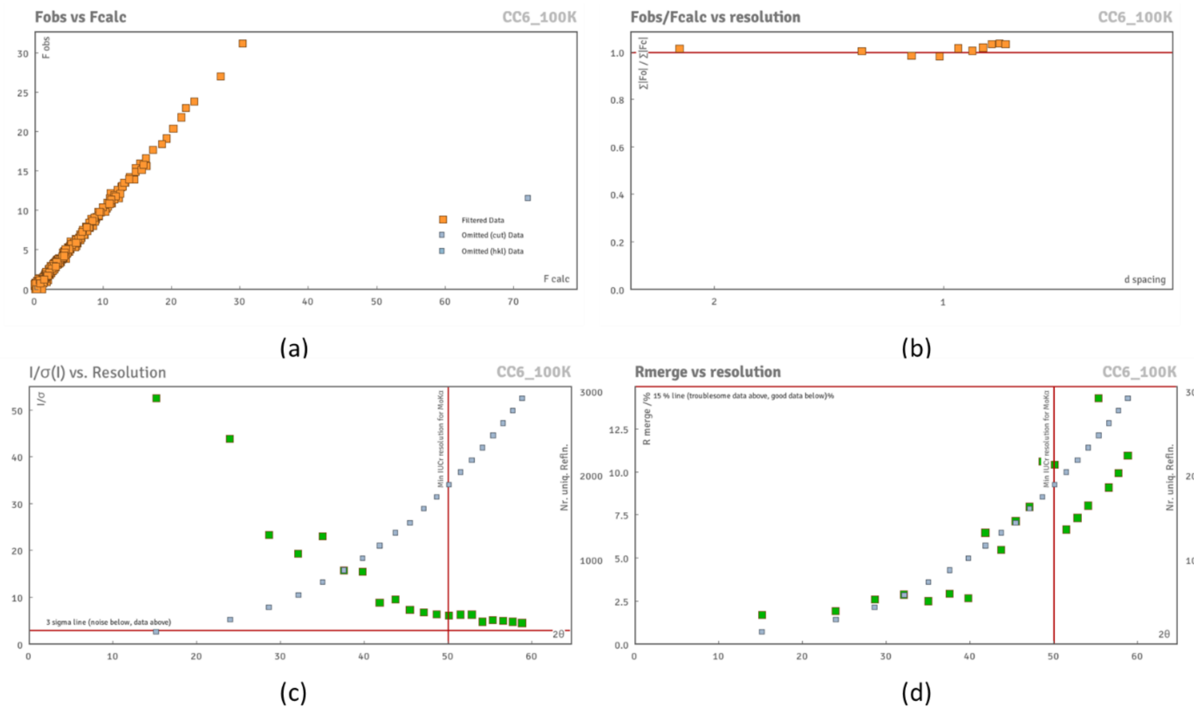

Structure **6**_100K

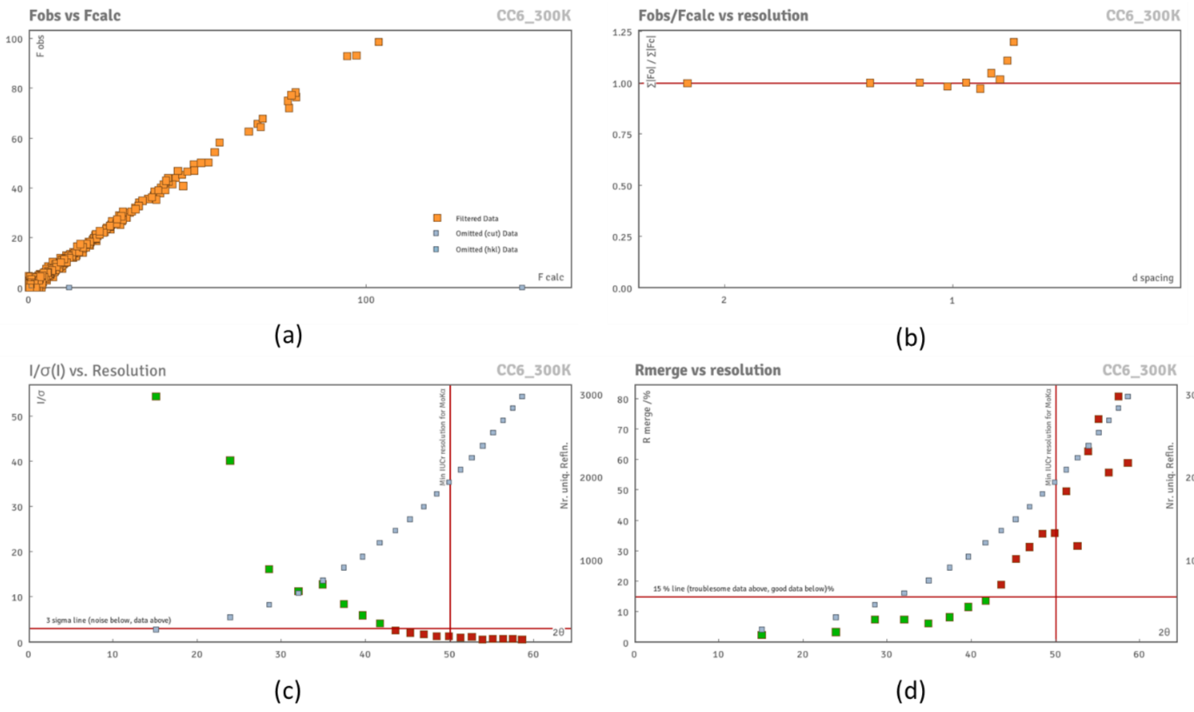

Structure **6**_300K

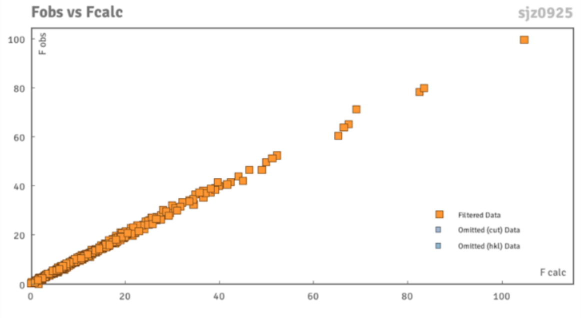

(a)

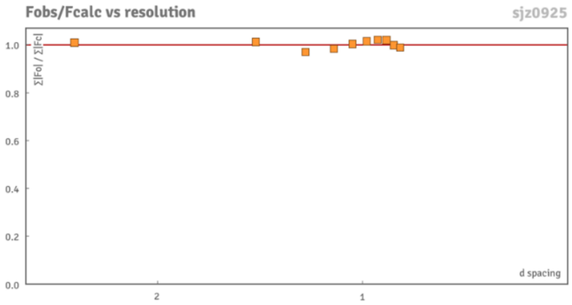

(b)

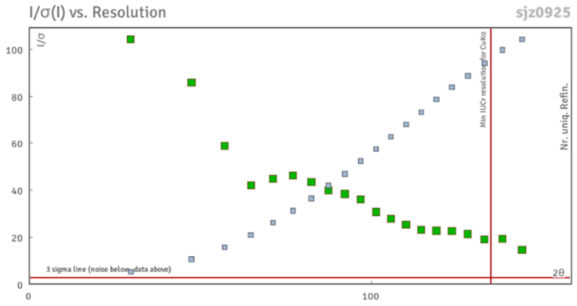

(c)

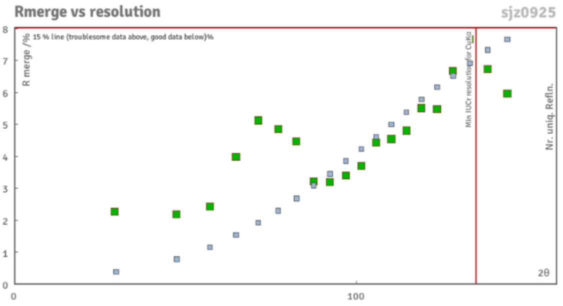

(d)

Structure **7**

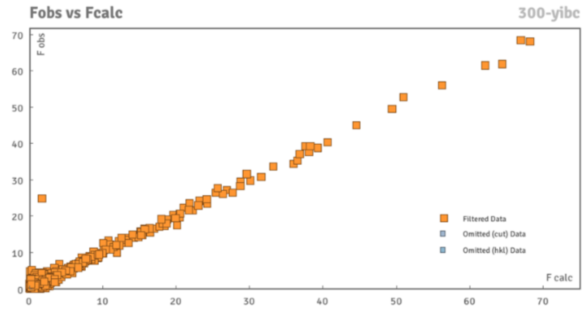

(a)

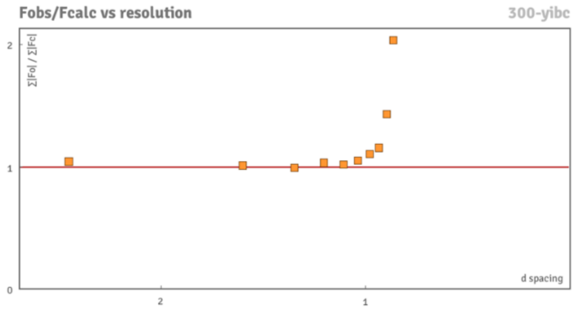

(b)

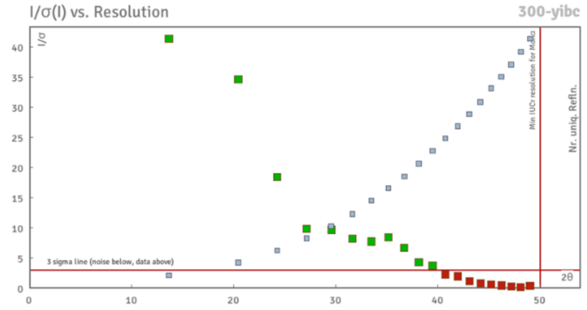

(c)

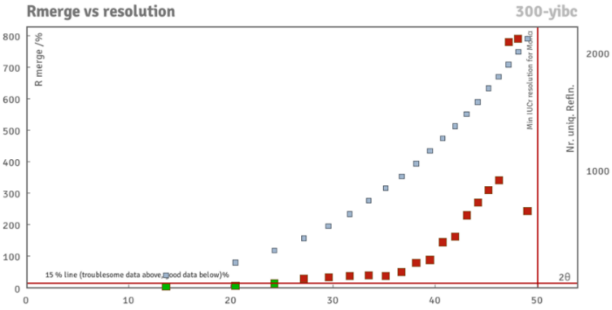

(d)

Structure **8**

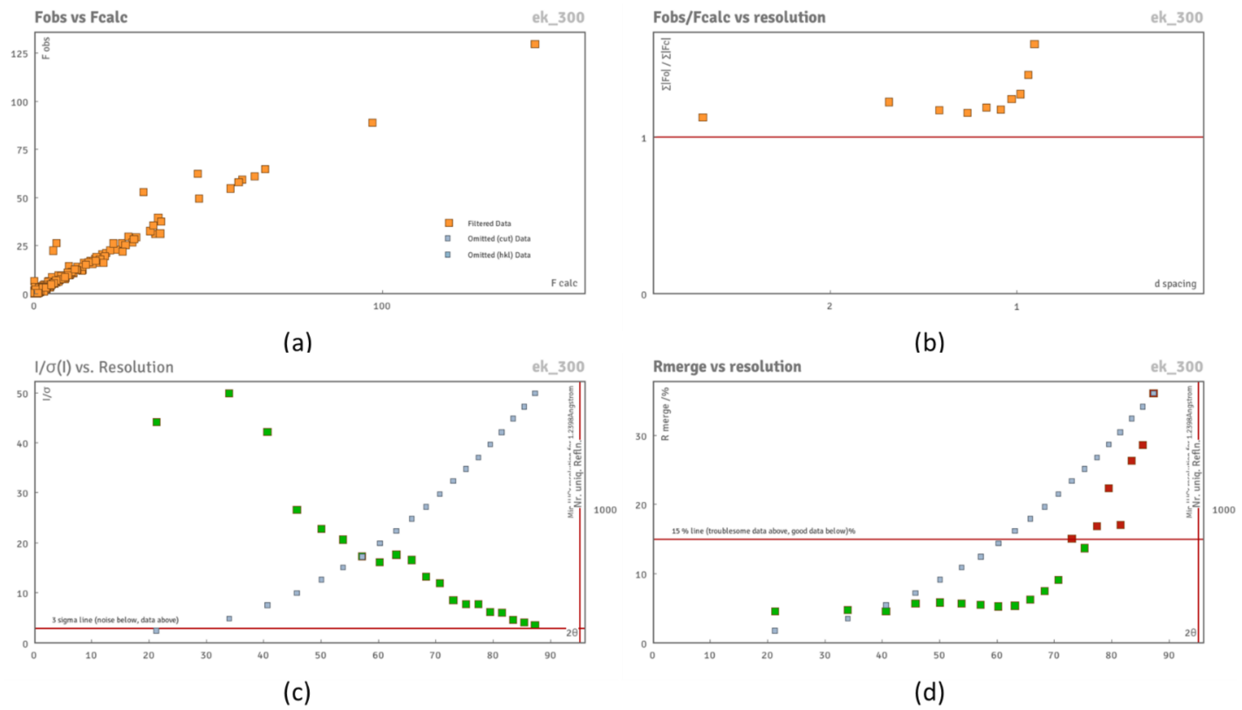

Structure **9**

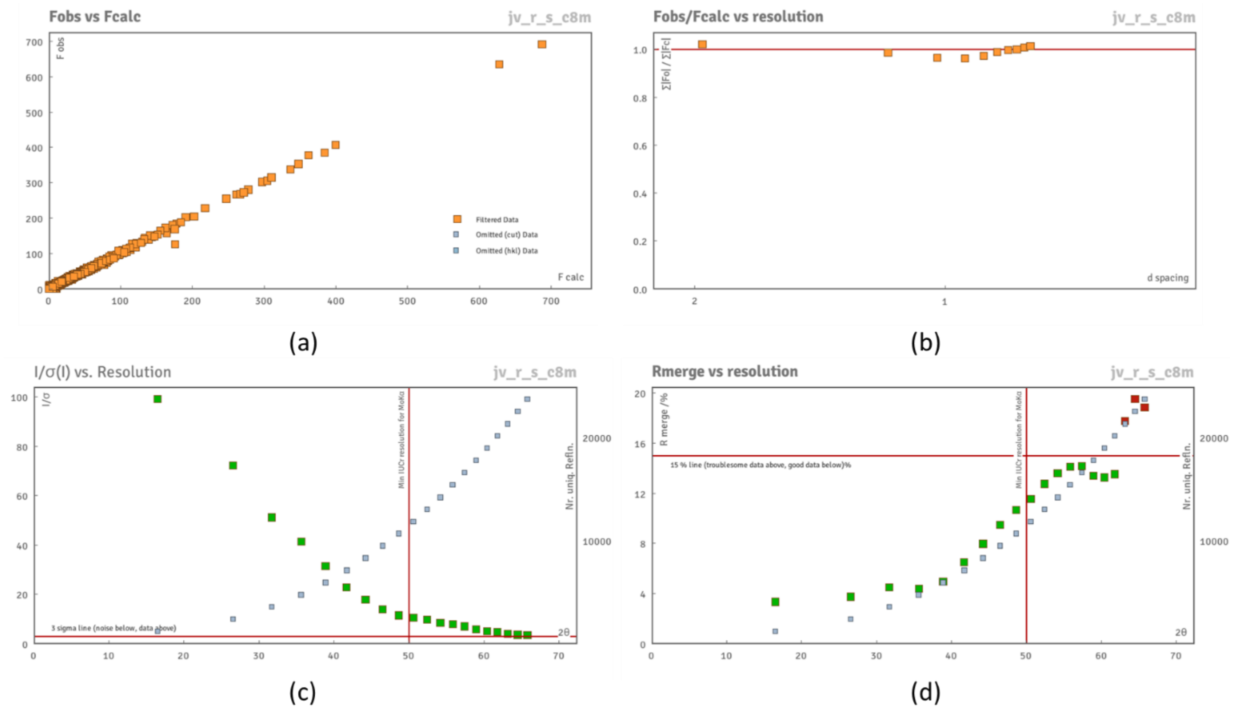

Structure **10**

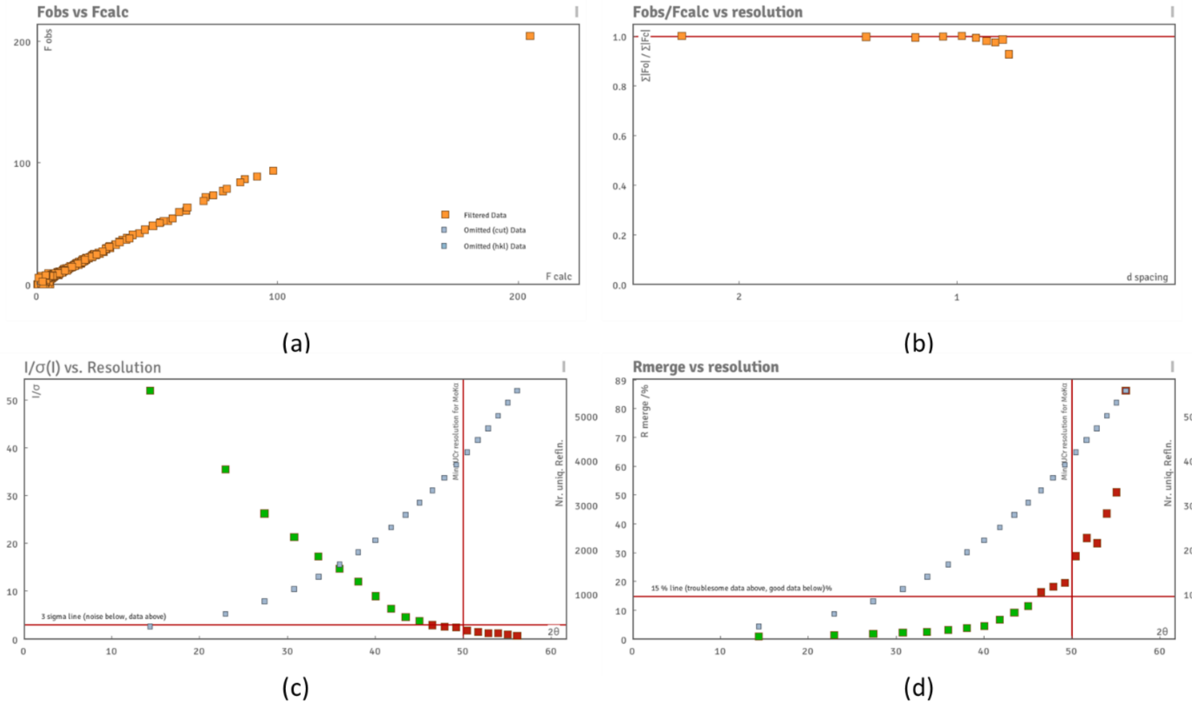

Structure **11**

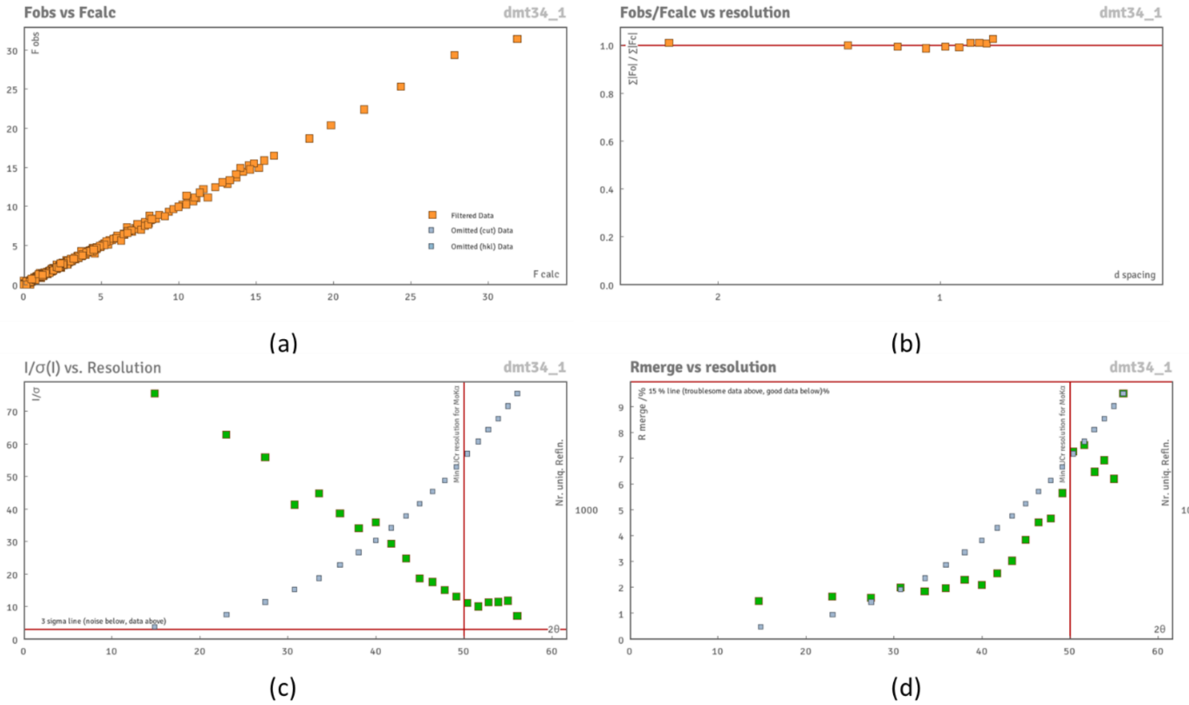

Structure **12**

Figure S1: Some data and model quality indicators for IAM refinements of structures **1-12** in olex2.refine: (a) Fobs vs Fcalc graph should be a straight line close to unity and any deviation indicates poor model or data quality, (b) Fobs/Fcalc (scale factor) vs resolution should be around 1 and any deviation indicates poor model or data quality, (c) I/σ(I) vs resolution graph shows binned data; if below 3σ(I) it indicates a high amount of noise and likely poor data quality, (d) Rmerge vs Resolution graph, in the cases where reflections were poor at high resolution the graph shows troublesome data. In structures **1** and **2** the data were merged.

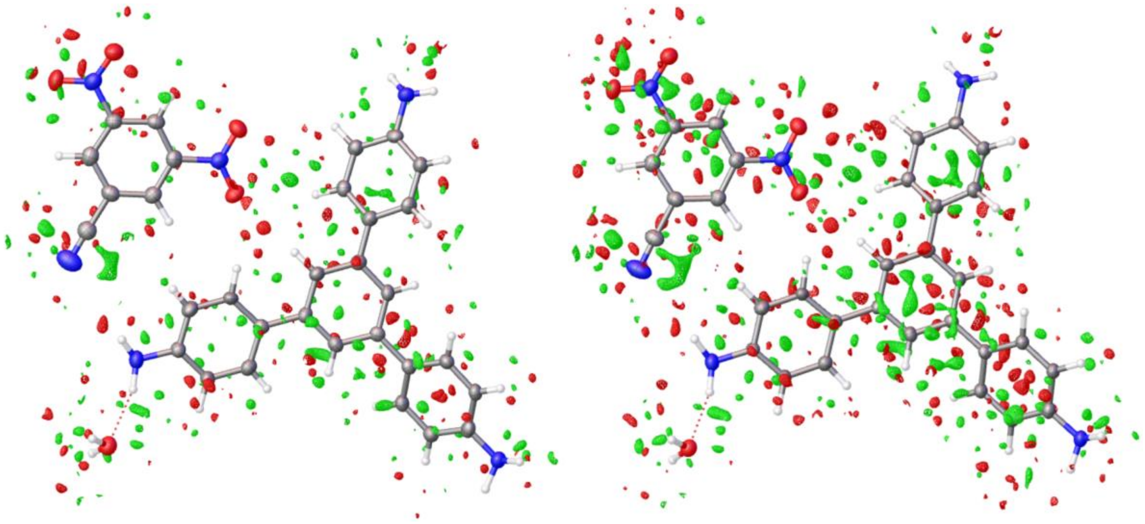

(a) IAM (b) TAAM

Figure S2: Comparison of residual density maps obtained for structure **3_100K** from IAM and TAAM. The contour level was set to 0.6 e Å^{-3}

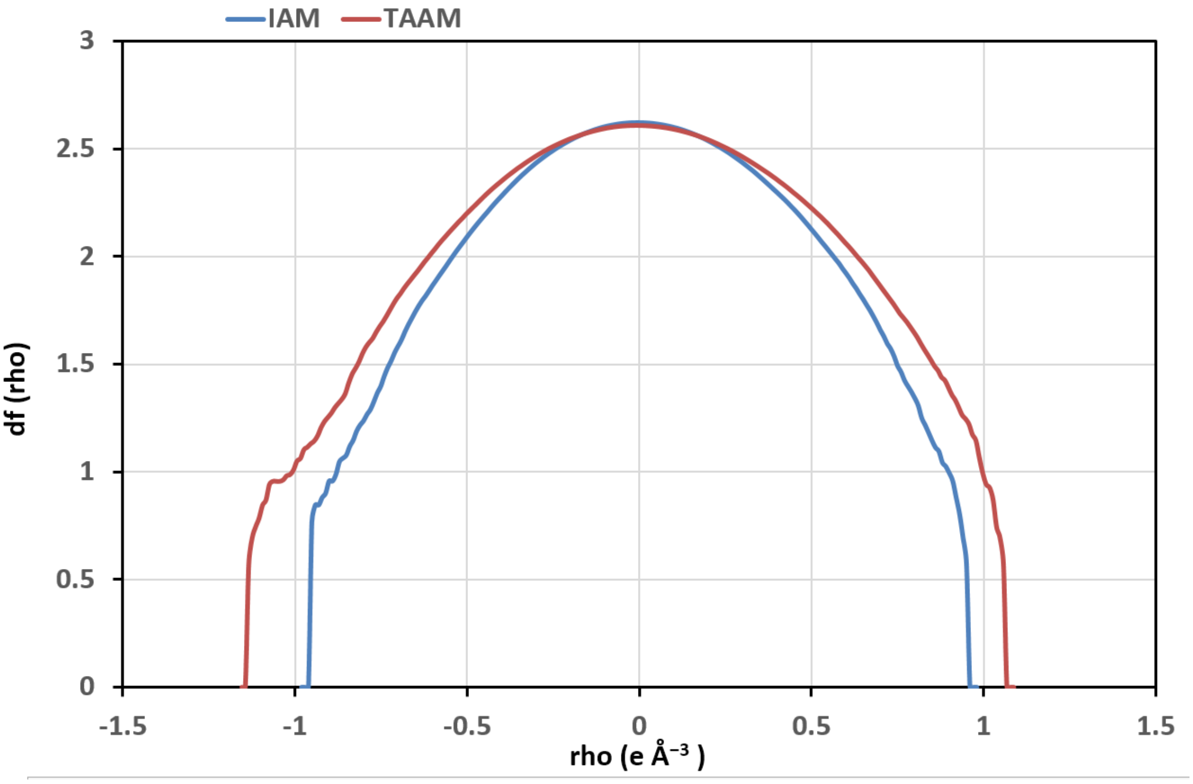

Figure S3: Comparison of fractal dimension plots for structure **3_100K** from IAM and TAAM.

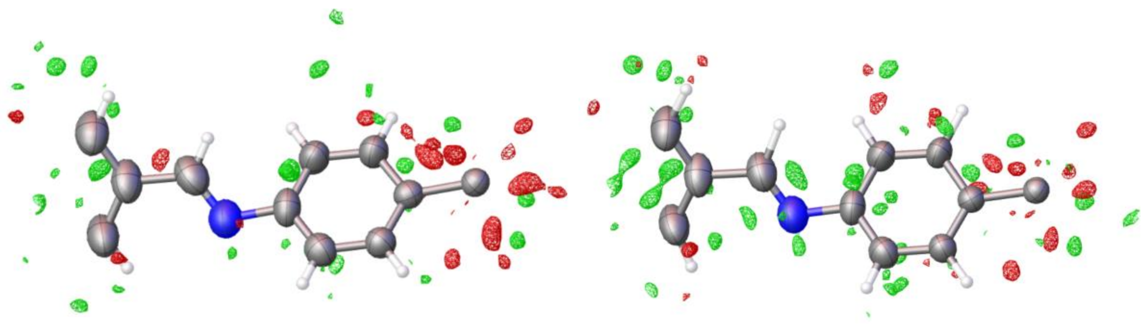

Figure S4: Comparison of residual density maps obtained for structure **8** from IAM and TAAM. The contour level was set to 0.25 e Å^{-3}

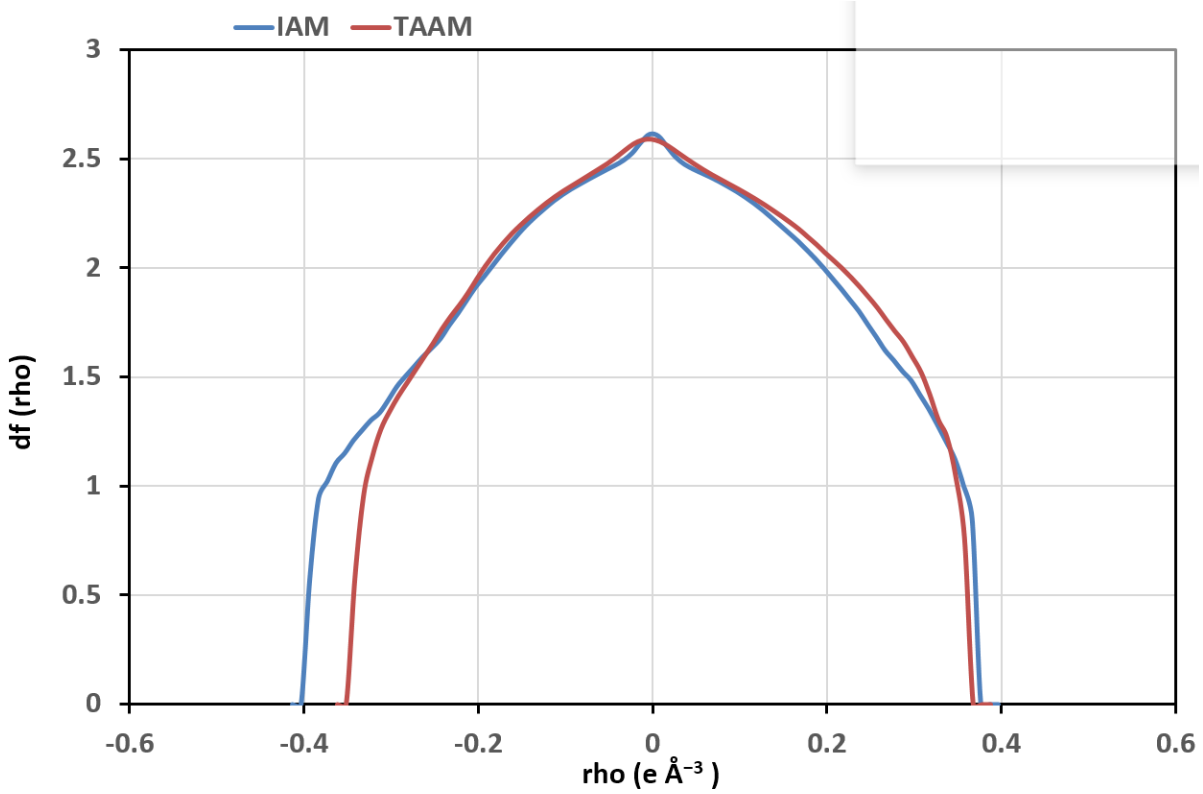

Figure S5: Comparison of fractal dimension plots for structure **8** from IAM and TAAM.

Table S1: Comparison of C-C bond precisions obtained from IAM and TAAM for structures **1**-**11**.

**Structure No.**	**IAM** C-C bond precision (Å)	**TAAM** C-C bond precision (Å)
**1**	0.0017	0.0011
**2**	0.0020	0.0011
$\mathbf{3}_{100K}$*	0.0066	0.0066
$\mathbf{3}_{300K}$*	0.0074	0.0070
$\mathbf{4}_{100K}$*	0.0018	0.0014
$\mathbf{4}_{300K}$*	0.0037	0.0033
$\mathbf{5}_{100K}$*	0.0020	0.0018

**5**$_{300K}$*	0.0032	0.0031
**6**$_{100K}$*	0.0020	0.0013
**6**$_{300K}$*	0.0020	0.0020
**7**	0.0043	0.0036
**7** ^{dm}	0.0019	0.0014
**8**	0.0043	0.0047
**9**	0.0038	0.0040
**10**	0.0016	0.0013
**10** ^{dm}	0.0016	0.0010
**11**	0.0054	0.0049

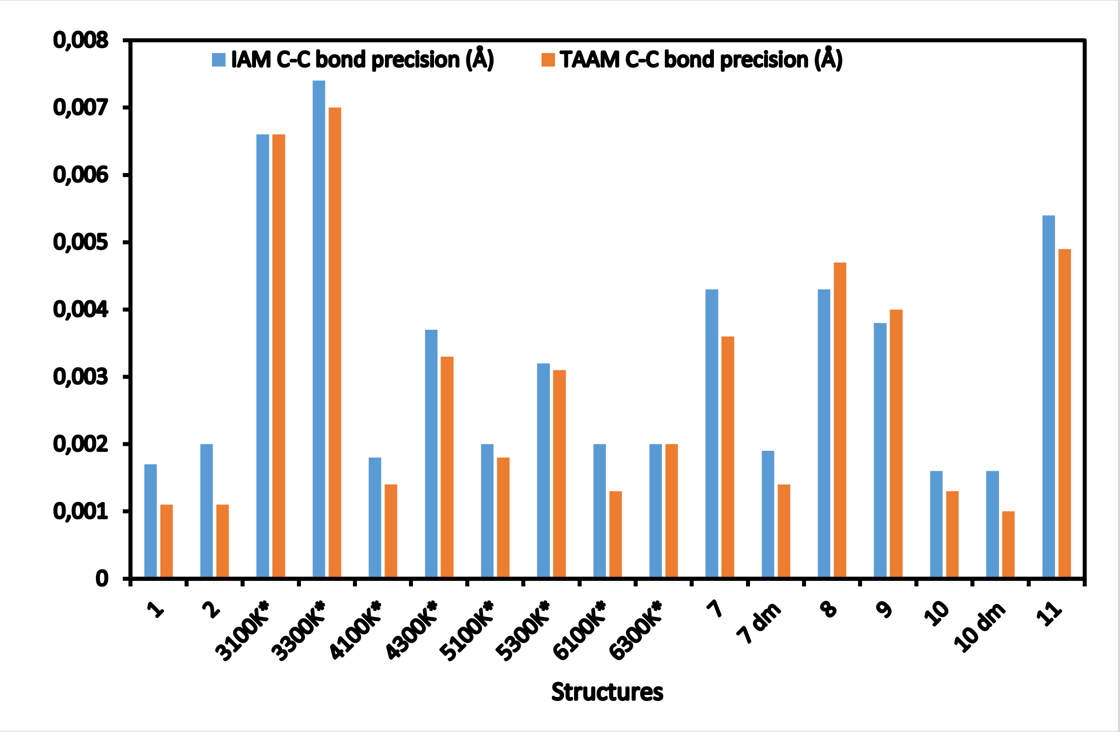

Figure S6: Comparison of C-C bond precisions obtained from IAM and TAAM for structures **1-11**.

Hybrid refinement details on structure 12

The coordinated metal-organic structure was divided into three parts: (a) PART 1 containing sodium atom (Na1), (b) PART 2 containing two water molecules, and (c) PART 3 containing the organic molecule as shown below.

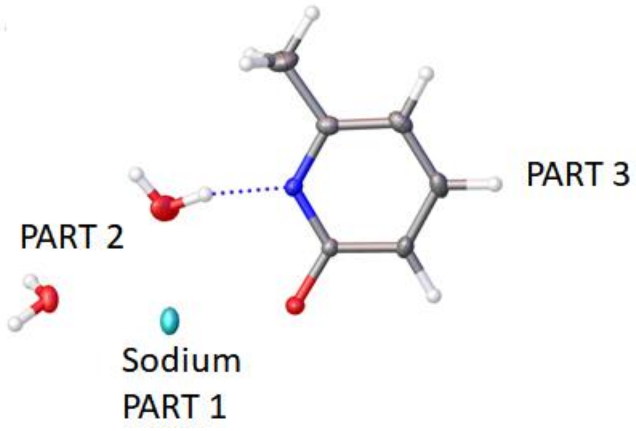

In the neutral IAM-TAAM hybrid refinement, no formal charges were assigned to sodium or the organic part. For IAM-TAAM and HAR-TAAM charge hybrid refinement, a charge of +1 e was assigned to sodium in PART 1 and -1 e to PART 3.

Table S2: Comparison of IAM, HAR, and TAAM refinement statistics for $\mathbf{3}_{100K}$. HAR was performed using a level of theory of B3LYP with different basis sets, once for a single cycle and iteratively until convergence was achieved. TAAM refinement was also performed for a single cycle and iteratively.

**Method**	**$R1_{gt}^{\#}$ (%)**	**$R1_{all}$ (%)**	**$wR2_{gt}^{\#}$ (%)**	**$wR2_{all}$ (%)**	**Peak/Hole (e Å^{-3})**	**Time**
olex2.refine IAM	9.25	20.82	18.00	23.82	0.97/ -0.94	~10 sec
3-21G	8.99	20.63	17.42	23.40	1.01/ -1.06	7 min
3-21G iterative	8.97	20.62	17.08	22.85	1.04/ -1.06	24 min
6-31G(d,p)	8.94	20.59	17.31	23.34	1.01/ -1.00	15 min
6-31G(d,p) iterative	8.91	20.57	16.92	22.72	1.01/ -0.96	59 min
Def2-TZVP	8.88	20.54	17.20	23.23	0.99/ -1.01	1h 21 min
Def2-TZVP iterative	8.84	20.52	16.61	22.29	0.99/ -1.00	4h 58 min
TAAM	8.94	20.57	17.35	23.36	1.00/ -0.97	~20 sec
TAAM iterative	8.93	20.58	17.03	22.85	1.07/ -1.03	2 min

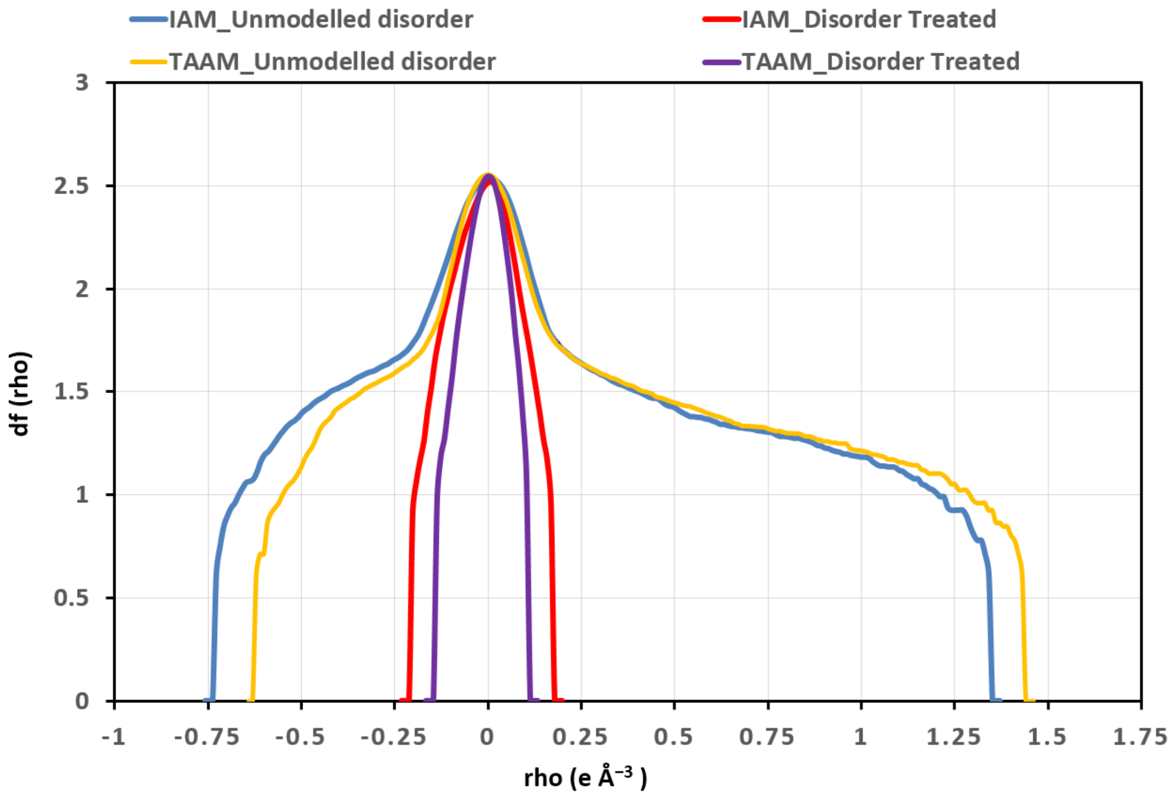

(a)

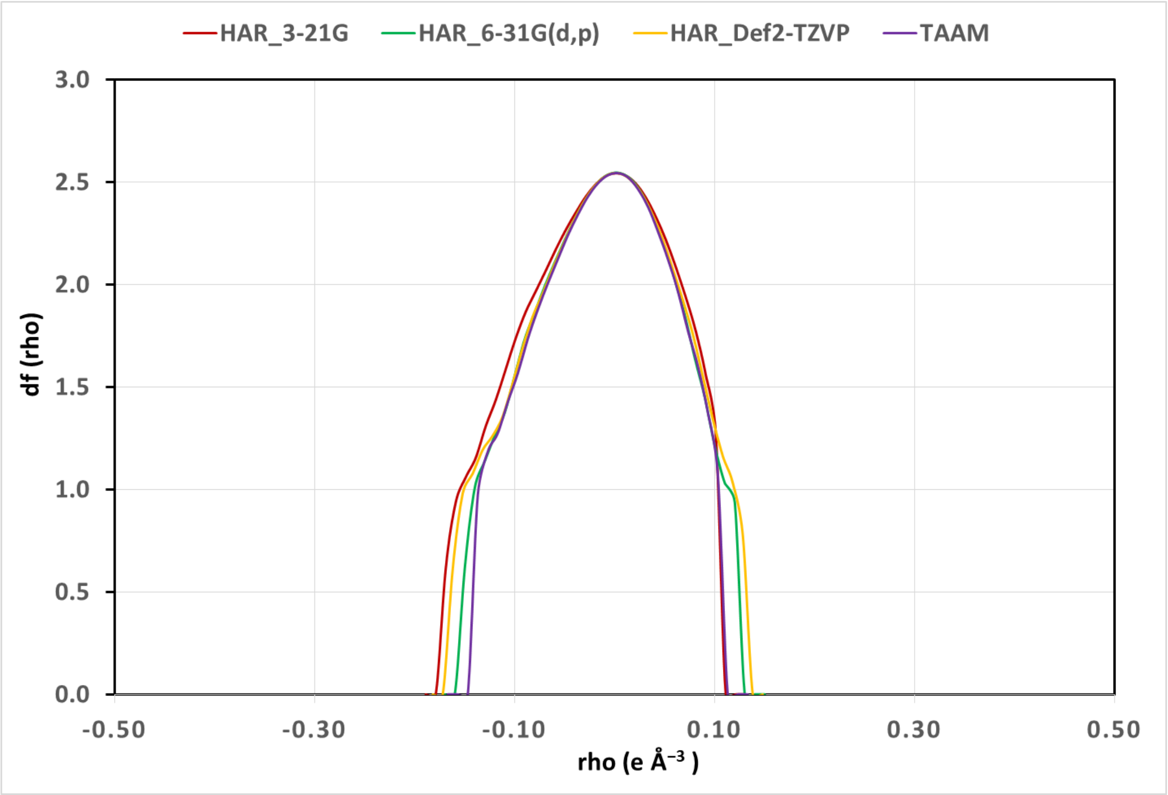

(b)

Figure S7: Comparison of fractal dimension plots (a) between IAM Reported disordered structure, IAM, and TAAM of disorder treated structure **7**, (b) between HAR with different basis sets, 3-21G, 6-31G(d,p), Def2-TZVP and TAAM of disorder treated structure **7**.

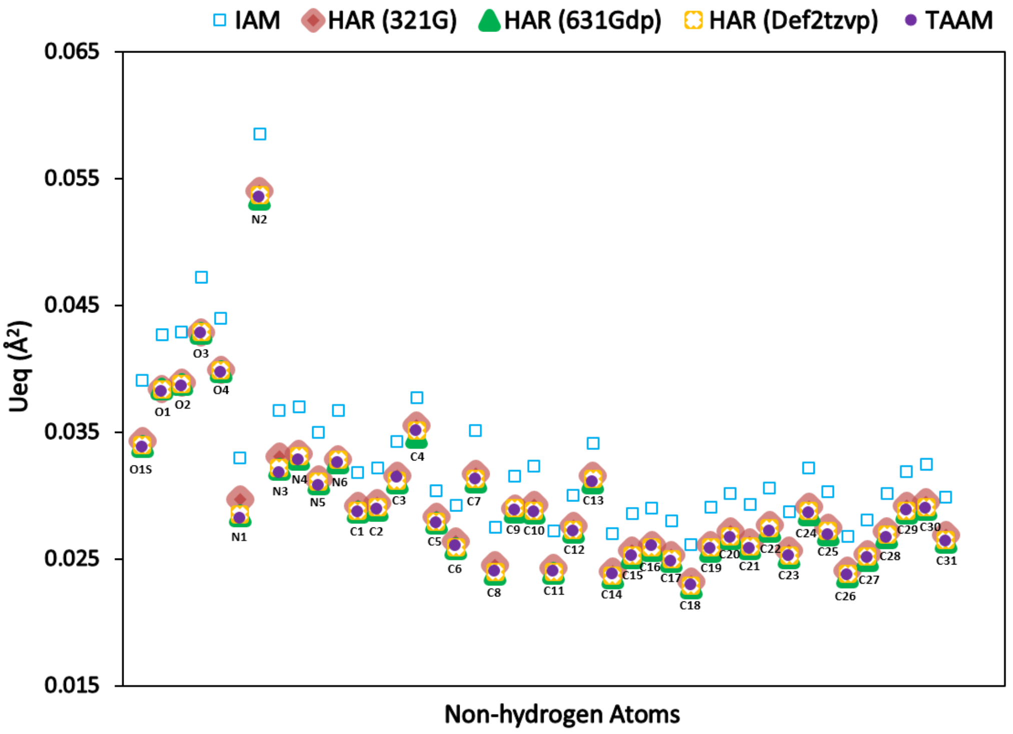

(a)

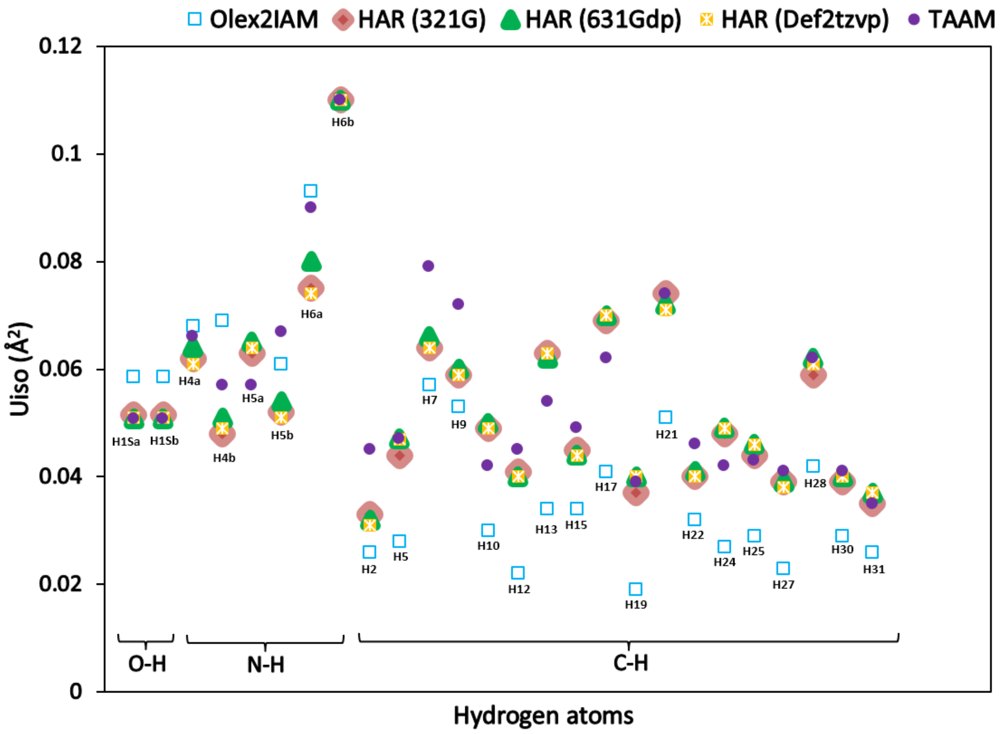

(b)

Figure S8: Comparison of displacement parameters of structure **3**$_{100K}$ after IAM, HAR, and TAAM refinements: (a) U_{eq} (Å^2) for non-hydrogen atoms (b) U_{iso} (Å^2) for hydrogen atoms.

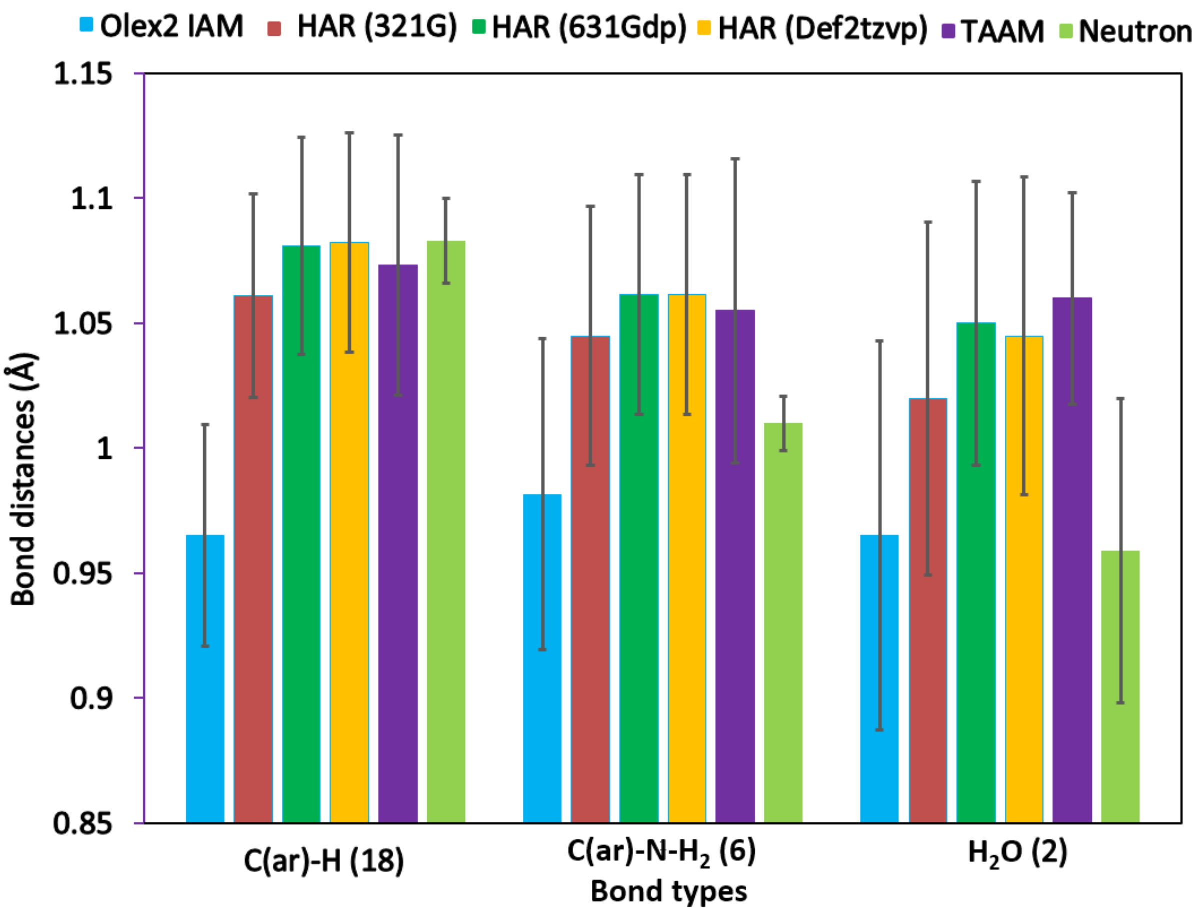

Figure S9: Comparison of X-H average bond lengths for structure **3**$_{100K}$ with neutron bond lengths as defined previously (Allen & Bruno, 2010). The O–H bonds in water molecules were referred to the corresponding neutron bond lengths were taken from Woinska *et. al*., 2016. The number in the parenthesis of the bond type indicates the number of occurrences.
